# miR156‐SPLs module regulates flowering and controls plant height by modulating gibberellin biosynthesis in citrus

**DOI:** 10.1111/pbi.70238

**Published:** 2025-06-30

**Authors:** Min Chen, Tian‐Liang Zhang, Wen‐Bo Zhang, Yong‐Zhen Wen, Zhong‐Xiang Ma, Zhen‐Ping Xi, Chun‐Gen Hu, Jin‐Zhi Zhang

**Affiliations:** ^1^ National Key Laboratory for Germplasm Innovation & Utilization of Horticultural Crops, College of Horticulture and Forestry Science Huazhong Agricultural University Wuhan 430070 China

**Keywords:** citrus, flowering, gibberellin biosynthesis, *miR156*, plant height

## Abstract

Plant height and optimal flowering time are key determinants of crop yield and economic value. However, the regulatory mechanisms governing these traits, particularly in woody plants, remain unclear. In this study, overexpression of a citrus *microRNA156* (*miR156*) family member, *Ci‐miR156c*, resulted in significant phenotypic changes in citrus, including reduced plant height and delayed flowering. *miR156*‐mediated repression of *SQUAMOSA PROMOTER‐BINDING PROTEIN‐LIKE* (*SPL*) genes is a highly conserved regulatory mechanism in plants. Yeast one‐hybrid and dual‐luciferase assays, along with other related experiments, indicated that the *Ci‐miR156c‐CiSPL7* module targets the bZIP transcription factor (*CiFD*) to regulate citrus flowering. Additionally, the *Ci‐miR156c‐CiSPL6* module regulates plant height by targeting *GA 20‐oxidase 2* (*CiGA20ox2*), a key gibberellin biosynthesis gene. The *Ci‐miR156c‐CiSPL3* module also influences plant height by regulating the KNOX family gene (*CiKN6*), which further regulates *CiGA20ox2* expression. Overexpression of *CiKN6* in citrus induced dwarfism, whereas its suppression increased height in transgenic plants, reinforcing its role in plant height regulation. Exogenous gibberellin and its inhibitor treatment further confirmed that the *miR156*‐*SPLs* module regulates citrus plant height by inhibiting gibberellin biosynthesis. These findings highlight the role of the *miR156‐SPLs* module in controlling citrus flowering and plant height.

## Introduction

Flowering represents the successful transition of plants from vegetative to reproductive growth. Optimal flowering time is essential for maximizing reproductive success and ensuring seed production, which are key steps in the evolutionary success of plants (Chen *et al*., 2023; Wu *et al*., 2009). However, long juvenile periods severely hamper the breeding of perennial woody plants (Wang *et al*., 2011). Developing methodologies for early flowering induction through trait identification and genetic manipulation could revolutionize breeding paradigms by enabling the effective utilization of advantageous recessive alleles and accelerating the accumulation of rare alleles via introgressive backcrossing strategies (Agustí *et al*., 2022; Wu *et al*., 2022). Furthermore, the traditional plant architecture of fruit trees, characterized by tall tree trunks and depressed crowns increases management costs. In contrast, dwarfing trees have a major impact on increasing planting density, improving photosynthetic efficiency, and facilitating harvesting (Hayat *et al*., 2021; Wang and Li, 2008). Therefore, the cultivation of dwarf fruit trees has emerged as a common practice in modern orchard management (Cheng *et al*., 2019). Recent advances in genome‐editing technologies have shown greater efficiency in developing dwarf cultivars with early flowering traits than conventional breeding methods (Cheng *et al*., 2019; Smolka *et al*., 2010). However, the molecular mechanisms underlying dwarfism and flowering regulation in perennial fruit trees, especially in citrus species, remain poorly characterized.

MicroRNA156 (*miR156*) is one of the most widespread and evolutionarily conserved miRNAs (Arazi *et al*., 2005), regulating plant development by repressing a subset of *SQUAMOSA PROMOTER BINDING PROTEIN‐LIKE* (*SPL*) genes (Axtell and Bowman, 2008). In *Arabidopsis*, the *miR156*‐*SPLs* module constitutes a novel age‐dependent flowering regulatory pathway among the known flowering pathways (Wang and Wang, 2015). This is evident as *miR156* is predominantly expressed in juvenile shoots but declines upon maturity, with lower *miR156* levels promoting adult‐phase development (Xie *et al*., 2012). Constitutive expression of *miR156* delays flowering and the *miR156*‐*SPLs* module functions downstream of *FLOWERING LOCUS T* (*FT*) and FD, a bZIP transcription factor (Wang *et al*., 2009). The complex of the mobile FT protein and FD plays a key role in activating genes that drive the transition from vegetative to reproductive development (Wang *et al*., 2009). Overexpression of *CiFT* and *CiFD* also results in early flowering in citrus, which is consistent with model plants (Wu *et al*., 2022; Ye *et al*., 2023). Additionally, *miR156* is highly conserved in different plants for the regulation of flowering; however, its effects on other aspects of plant development differ. For example, *miR156* negatively regulates nodulation in *Lotus japonicus* (Wang *et al*., 2015) and tuberization in potato (Bhogale *et al*., 2014). Previous studies have also shown that the *miR156*‐*SPLs* module controls male fertilization (Xing *et al*., 2010), leaf trichome development (Yu *et al*., 2010), and anthocyanin biosynthesis (Gou *et al*., 2011) in *Arabidopsis*. However, whether *miR156* is involved in the regulation of plant height and flowering in citrus remains unclear.

Plant height is regulated by complex interactions among phytohormones, key transcription factors, and environmental signals (Barbier *et al*., 2019; Hollender and Dardick, 2015). Among the major hormonal regulators, gibberellin (GA), brassinosteroid (BR), auxin (IAA), and cytokinin (CTK) have been identified as key players in modulating stem elongation and overall plant architecture (Hayat *et al*., 2021). GA, in particular, plays a key role in the regulation of plant height (Coles *et al*., 1999; Yamaguchi, 2008). GA promotes internode growth and increases plant height by stimulating cell division and elongation during plant development (Miao *et al*., 2020; Yamaguchi, 2008). GA biosynthesis is catalysed by seven key enzymes: *ent*‐copalyl diphosphate synthase (CPS), *ent*‐kaurene synthase (KS), *ent*‐Kaurene oxidase (KO), *ent*‐kaurenoic acid oxidase (KAO), GA 20‐oxidase (GA20ox), GA 3‐oxidase (GA3ox), and GA 2‐oxidase (GA2ox) (Yamaguchi, 2008). As central regulators of the GA pathway, mutations in GA biosynthesis genes, such as *GA20ox* (Plackett *et al*., 2012) and *GA3ox* (Chen *et al*., 2014), lead to plant dwarfism by reducing endogenous GA levels, resulting in the accumulation of DELLA proteins that limit internode elongation. For example, overexpression or downregulation of *GA20ox* modified plant height by altering the concentrations of GA in *Arabidopsis* (Coles *et al*., 1999), hybrid aspen (Eriksson *et al*., 2000), tobacco (Vidal *et al*., 2001), apple (Bulley *et al*., 2005), and citrus (Fagoaga *et al*., 2007; Vidal *et al*., 2001). However, the molecular regulatory mechanisms underlying plant height in citrus plants still remains poorly understood.

In plants, the shoot apical meristem (SAM) is a dome‐shaped structure situated at the aerial growth tip of a shoot that harbours stem cells (Luo *et al*., 2024). These stem cells produce daughter cells that differentiate into the above‐ground organs (Luo *et al*., 2024), making the development and maintenance of the SAM crucial for plant morphology and growth (Narnoliya *et al*., 2019). Previous studies have demonstrated a significant relationship between internode development and the SAM (Liu *et al*., 2022), with *KNOTTED‐ LIKE HOMEODOMAIN* (*KNOX*) genes playing key roles in the establishment, organogenesis, and maintenance of SAM (Su *et al*., 2020; Tsuda *et al*., 2011). The developmental functions of *KNOX* genes are closely linked to various plant hormones (Barth *et al*., 2009). For instance, in maize, *KNOTTED1* directly activates *GA2ox1* (Bolduc and Hake, 2009), while in tobacco, KNOX protein homeobox 15 negatively regulates *GA20ox* expression in the SAM, leading to reduced levels of bioactive GA (Sakamoto *et al*., 2001). Similarly, in *Arabidopsis*, *KNOX* regulates hormone homeostasis by repressing GA biosynthesis and activating CTK to promote meristem activity in the SAM (Jasinski *et al*., 2005). A previous study showed that the *SPL11* gene might be related to the development of citrus leaves by targeting the *KNOX* gene (Zeng *et al*., 2022). Moreover, *KNOX* genes have been instrumental in understanding various developmental processes, including delimitation of leaf primordia (Piazza *et al*., 2010), establishment of inflorescence architecture (Li *et al*., 2012), and internode development (Tsuda *et al*., 2017).

Citrus is globally recognized as one of the most economically significant fruit crops (Wu *et al*., 2018). In modern citrus cultivation, dwarfism and early flowering have become critical agronomic traits that enable high‐density plantations to improve the efficiency of orchard management (Agustí *et al*., 2022). Currently, the primary method for controlling plant height and flowering during citrus production involves grafting onto specific rootstocks (Amancio *et al*., 2017). Therefore, understanding the mechanisms underlying citrus dwarfism and flowering is essential for the development and utilization of optimal rootstock varieties. To identify flowering‐related miRNAs, several small RNA libraries were constructed from an early flowering trifoliate orange mutant (precocious trifoliate orange, *Citrus trifoliata* L. Raf.), leading to the discovery of several novel and conserved flowering‐related miRNAs, including members of the *miR156* and *miR172* families (Sun *et al*., 2012; Zhang *et al*., 2012). In this study, the ectopic expression of *Ci‐miR156c* resulted in a late flowering and dwarf phenotype in tobacco. The overexpression of *Ci‐miR156c* in trifoliate orange resulted in late flowering and significantly reduced plant height. Further studies revealed that the *Ci‐miR156c*‐*CiSPLs* module regulates plant height and flowering by targeting *CiKN6* and *CiGA20ox2*, and *CiFD*, respectively. This study provides valuable insights into the mechanisms underlying the regulation of plant height and flowering, particularly in woody plants.

## Results

### *Ci‐miR156c* is involved in the regulation of flowering and plant height

To identify miRNAs related to citrus flowering, three *miR156* precursors (*Ci‐MIR156a*, *Ci‐MIR156b*, and *Ci‐MIR156c*) were previously identified through genome‐wide deep sequencing, and their expression patterns were closely associated with flowering (Sun *et al*., 2012; Zhang *et al*., 2012). Sequence analysis revealed significant differences in the precursor sequences of *Ci‐MIR156a*, *Ci‐MIR156b*, and *Ci‐MIR156c* (Figure S1). The mature sequence of *Ci‐miR156* was highly similar to that of other plants (Figure S2). To explore their functions, the three precursors (*Ci‐MIR156a*, *Ci‐MIR156b*, and *Ci‐MIR156c*) were ectopically expressed in tobacco. A total of 6, 8, and 9 transgenic lines were obtained for *Ci‐MIR156a*, *Ci‐MIR156b*, and *Ci‐MIR156c*, respectively. Two independent transgenic lines of each precursor were randomly selected for phenotypic analysis. Compared with the controls, *Ci‐miR156a*‐overexpressing tobacco exhibited no significant phenotypic differences (Figure S3). In contrast, both *Ci‐miR156b* and *Ci‐miR156c* transgenic tobacco displayed a delayed flowering phenotype compared with the controls (Figure S4a,b). Notably, *Ci‐miR156c* tobacco exhibited a greater delay in flowering than *Ci‐miR156b* transgenic tobacco.

To further explore the regulatory functions of *Ci‐miR156b* and *Ci‐miR156c* in flowering, we analysed the expression of key tobacco flowering‐related genes, *NtFT*, *LEAFY* (*NtLFY*), and *APETALA1* (*NtAP1*), in *Ci‐miR156b* and *Ci‐miR156c* transgenic tobacco. In *Ci‐miR156b* transgenic plants, *NtLFY* expression was significantly suppressed compared with that in the controls, whereas *NtFT* and *NtAP1* levels were not significantly different (Figure S4f–h). Compared with the controls, the expression of the three genes was significantly downregulated in *Ci‐miR156c* transgenic tobacco (Figure S4f–h).

**Figure 1:**
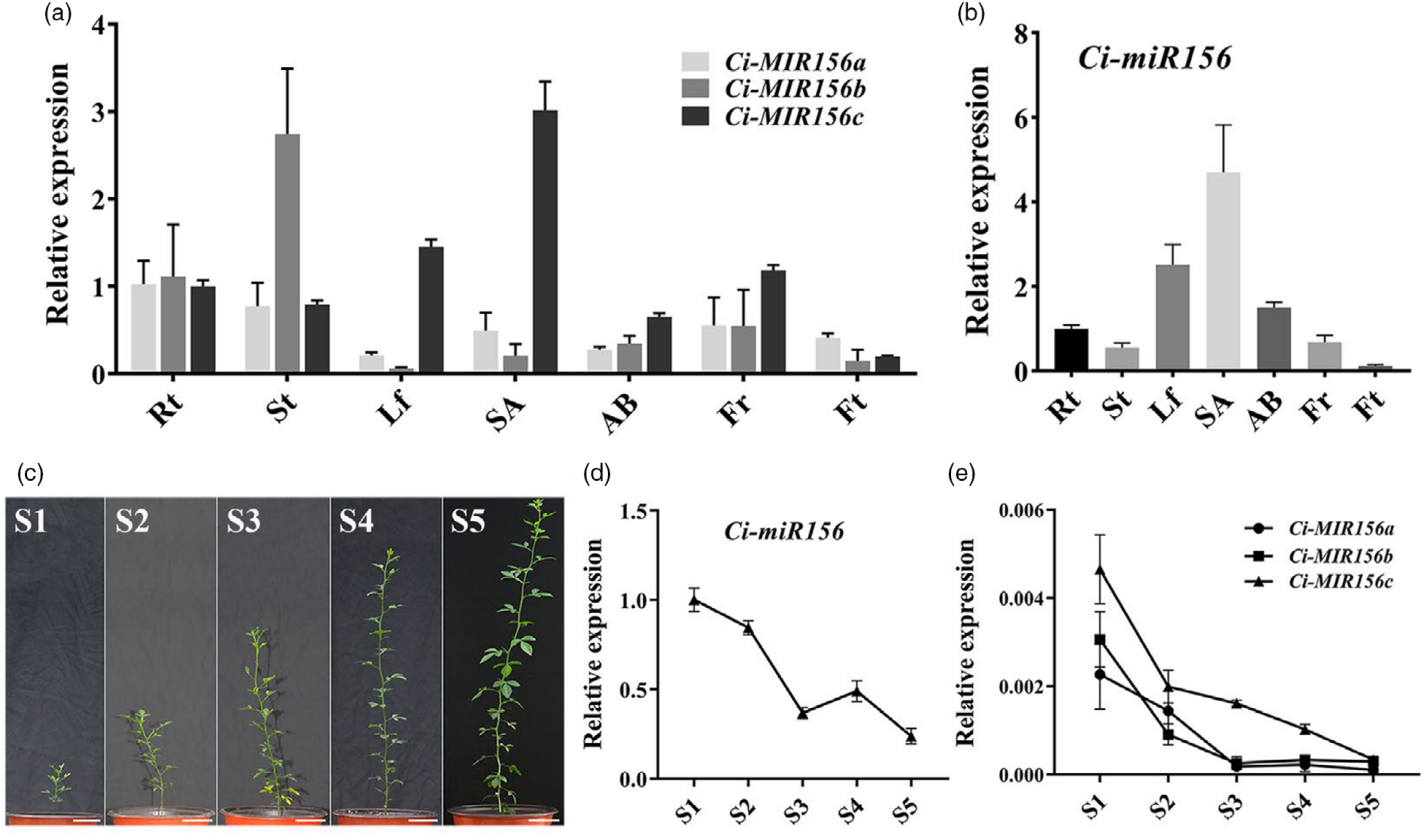
The expression analysis of *Ci‐miR156* in trifoliate orange. (a) The expression of *Ci‐MIR156a*, *Ci‐MIR156b*, and *Ci‐MIR156c* in various tissues of adult trifoliate orange. AB, axillary bud from spring shoot; Fr, flower at full bloom; Ft, fruit (young fruit from 30 days after flowering); Lf, healthy mature leaf; Rt, lateral root; SA, shoot apex from spring shoot; St, stem from spring shoot. (b) The expression of mature *Ci‐miR156* in various tissues of adult trifoliate orange. Rt was used as the control (with relative expression level set as 1.0). (c) Different developmental stages of plant height in trifoliate orange. S: stage, from S1 to S5 represents different development stages with increasing plant height. Scale bar = 5 cm. (d) The expression of mature *Ci‐miR156* in the shoot apex during plant height development. S1 was used as the control (with relative expression level set as 1.0). (e) The expression of *Ci‐MIR156a*, *Ci‐MIR156b*, and *Ci‐MIR156c* in the shoot apex during plant height development. The expression level of reference gene was used as the control. Citrus *U6* was used as the internal reference gene. Data represent means ± SE (*n* = 3).

In addition, *Ci‐miR156b* and *Ci‐miR156c* transgenic tobacco exhibited a dwarfism phenotype compared to the controls (Figure S4a). Further statistical analysis showed that plant height and average internode length of *Ci‐miR156b* and *Ci‐miR156c* transgenic tobacco were significantly reduced compared to those of the controls, but no significant difference was observed in leaf number (Figure S4c–e). Notably, the phenotype of *Ci‐miR156c* transgenic tobacco was more dwarfing than that of *Ci‐miR156b*, accompanied by shorter plant height and internode length. Tissue‐specific expression analysis of the three *miR156* precursors revealed that the *Ci‐MIR156c* precursor was mainly expressed in the shoot apex, whereas the precursors *Ci‐MIR156a* and *Ci‐MIR156b* were highly expressed in the root and stem, respectively (Figure 1a). Further expression analysis of mature *Ci‐miR156* showed that it was primarily expressed in the shoot apex, leaf, and axillary bud (Figure 1b), similar to the expression pattern of the *Ci‐MIR156c* precursor. These results suggested that the precursor *Ci‐MIR156c* may be the main source of mature *Ci‐miR156* expression. To further investigate the relationship between *Ci‐miR156* and plant height, the expression of *Ci‐miR156* was analysed in the shoot apex of trifoliate orange plants at different heights. The results showed that the expression of mature *Ci‐miR156* was gradually downregulated with increasing plant height (Figure 1c,d). To determine the precursor that plays a key role in the development of dwarfism, the expression of the three *miR156* precursors was investigated. The results showed that the expression of the three precursors was downregulated with increasing plant height; however, the expression level of *Ci‐MIR156c* was higher than that of *Ci‐MIR156a* and *Ci‐MIR156b* (Figure 1e). These results suggested that *Ci‐miR156c* plays an important role in flowering and plant height regulation; therefore, it was selected for further research.

### Phenotypic analysis of *Ci‐miR156c*‐OE transgenic citrus

**Figure 2:**
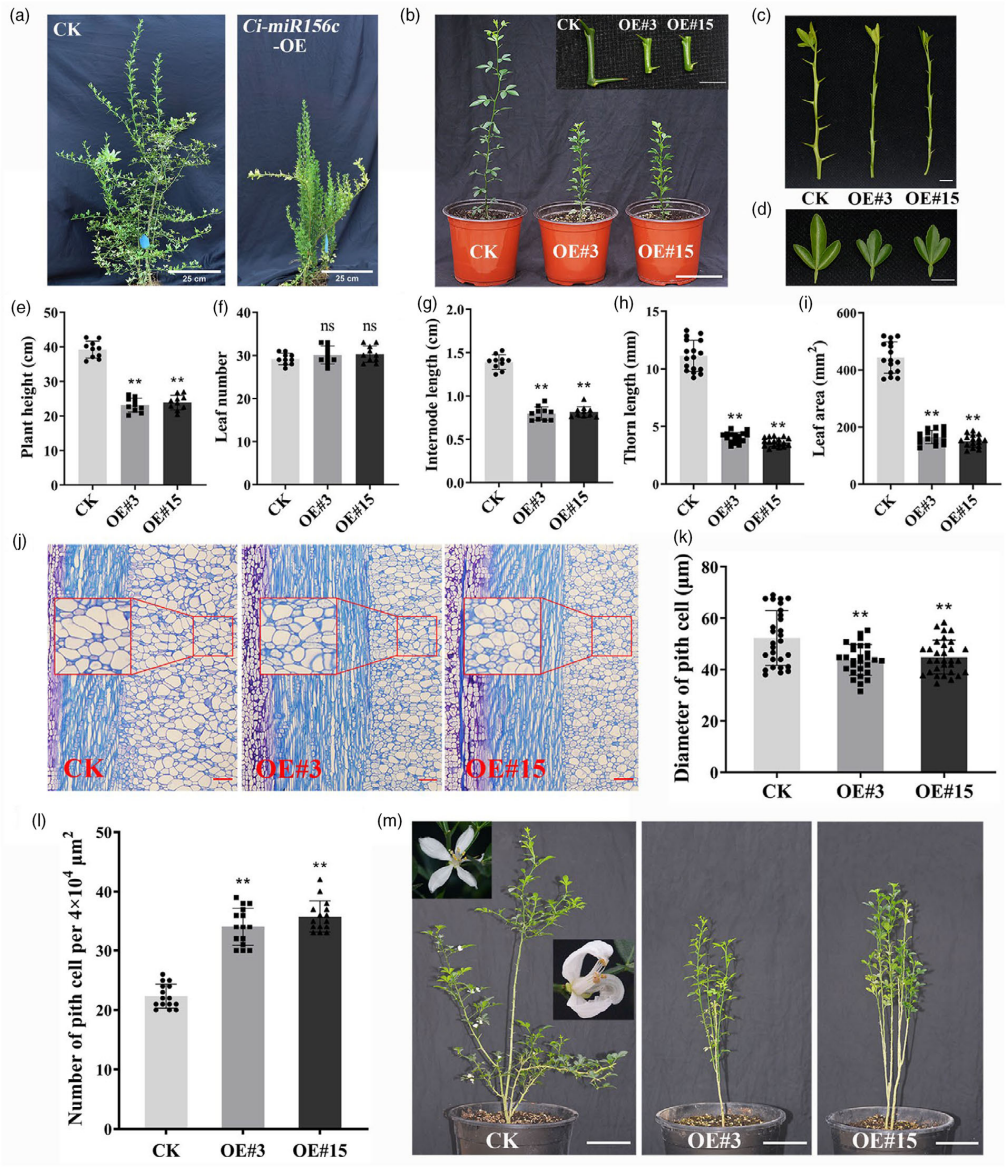
Phenotype analysis of *Ci‐miR156c*‐OE (overexpression) transgenic citrus. (a) Phenotypic characteristics of 3‐year‐old *Ci‐miR156c*‐OE transgenic trifoliate orange, scale bar = 25 cm. (b) Phenotypic characteristics of 8‐month‐old *Ci‐miR156c*‐OE transgenic trifoliate orange. CK represents the control. OE#3 and OE#15 represent two *Ci‐miR156c‐*OE transgenic lines. Scale bar = 10 cm. (c) Phenotypic analysis of the thorn length in 8‐month‐old *Ci‐miR156c*‐OE transgenic lines. Scale bar = 1 cm. (d) Phenotypic analysis of the leaf area in 8‐month‐old *Ci‐miR156c*‐OE transgenic lines. Scale bar = 1 cm. (e–i) Statistical analysis of plant height (e), leaf number (f), internode length (g), thorn length (h), and leaf area (i) in 8‐month‐old *Ci‐miR156c‐*OE transgenic trifoliate orange compared with the control. Data represent means ± SE (*n* ≥ 10). (j) Longitudinal section of the 5th internode (from top to bottom) of 8‐month‐old control and *Ci‐miR156c‐*OE transgenic trifoliate orange. Scale bar = 100 μm. The red boxes indicate randomly selected areas for subsequent statistical analysis of the diameter of pith cells and the number of pith cells. (k) Statistical analysis of the diameter of pith cells in longitudinal section. Data represent means ± SE (*n* ≥ 30). (l) Statistical analysis of the number of pith cells per 4 × 10^4^ μm^2^ in longitudinal section. Data represent means ± SE (*n* = 15). (m) Phenotypic analysis of flowering‐related traits of 2‐year‐old *Ci‐miR156c*‐OE transgenic trifoliate orange compared with the control. Scale bar = 10 cm. Statistically significant differences compared to the control are marked with asterisks (*P < 0.05, **P < 0.01, ns indicates no significant difference, Student's *t*‐test).

To investigate the function of *Ci‐miR156c* in citrus, 14 independent *Ci‐miR156c* overexpression (OE) transgenic lines of trifoliate orange were generated. Phenotypic observation showed that the *Ci‐miR156c*‐OE plants were dwarfed compared with the control plants (Figure 2a). Subsequently, two transgenic lines (#3 and #15) from the *Ci‐miR156c*‐OE transgenic trifoliate orange were randomly selected for further phenotypic analyses (Figure 2b). Compared with the controls, the expression levels of *Ci‐MIR156c* and *Ci‐miR156* were significantly upregulated (fivefold or higher) in the transgenic lines (Figure S5a,b). Consistent with observations in tobacco, *Ci‐miR156c*‐OE transgenic trifoliate orange lines exhibited a dwarf phenotype with shorter internodes than the controls (Figure 2b). Additionally, thorn length and leaf area were reduced in the *Ci‐miR156c*‐OE lines (Figure 2c,d). Statistical analysis of plant height, leaf number, average internode length, thorn length, and leaf area revealed that the overexpression lines exhibited significant reductions in plant height, average internode length, thorn length, and leaf area compared to the controls, whereas no significant differences were observed in leaf number (Figure 2e–i). Paraffin section analysis of longitudinal internode section revealed that the pith cell diameter in *Ci‐miR156c*‐OE lines was significantly smaller than that in the controls, and the number of pith cells was increased (Figure 2j–l). Further investigation revealed that the leaf epidermal cell size of control plants was larger than that of *Ci‐miR156c*‐OE plants, suggesting that leaf size reduction may be associated with decreased cell expansion (Figure S6). In addition to the dwarf phenotype, *Ci‐miR156c*‐OE transgenic trifoliate oranges also exhibited delayed flowering compared with the controls (Figure 2m).

### Phenotype analysis of *Ci‐miR156c*‐STTM transgenic citrus

**Figure 3:**
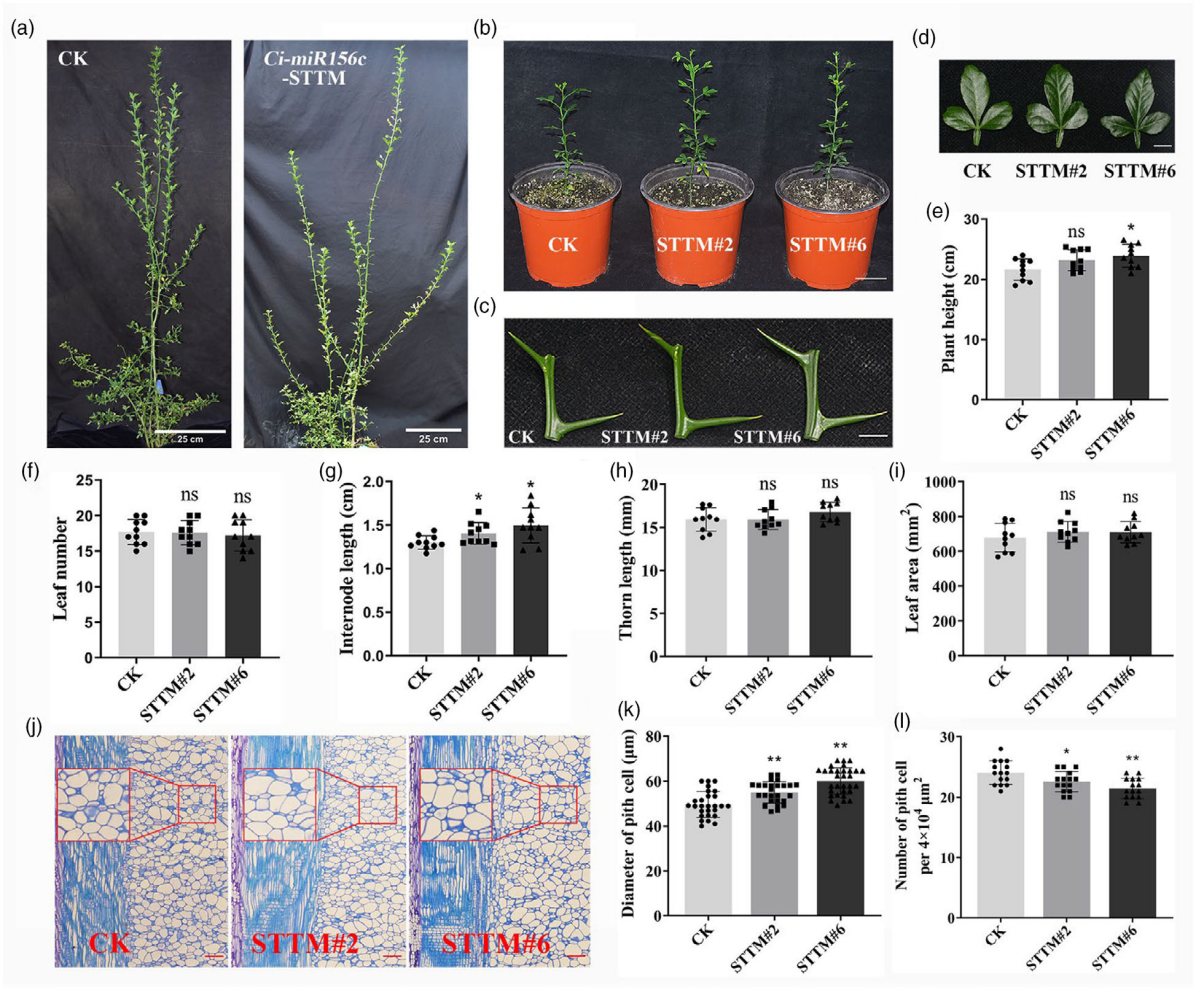
Phenotype analysis of *Ci‐miR156c*‐STTM transgenic citrus. (a) Phenotypic characteristics of 3‐year‐old *Ci‐miR156c*‐STTM transgenic trifoliate orange compared with the control, scale bar = 25 cm. (b) Phenotypic characteristics of 6‐month‐old *Ci‐miR156c*‐STTM transgenic trifoliate orange. CK represents the control. STTM#2 and STTM#6 represent two *Ci‐miR156c*‐STTM transgenic lines. Scale bar = 5 cm. (c) Comparative analysis of the internode and thorn length between 6‐month‐old *Ci‐miR156c*‐STTM transgenic trifoliate orange and control. Scale bar = 1 cm. (d) Phenotypic analysis of the leaf area in 6‐month‐old *Ci‐miR156c*‐STTM transgenic lines. Scale bar = 1 cm. (e–i) Statistical analysis of plant height (e), leaf number (f), internode length (g), thorn length (h), and leaf area (i) in 6‐month‐old *Ci‐miR156c*‐STTM transgenic trifoliate orange compared with the control. Data represent means ± SE (*n* = 10). (j) Longitudinal section of the 5th internode (from top to bottom) of 6‐month‐old control and *Ci‐miR156c*‐STTM transgenic trifoliate orange. Scale bar = 100 μm. The red boxes indicate randomly selected areas for subsequent statistical analysis of the diameter of pith cells and the number of pith cells. (k) Statistical analysis of the diameter of pith cells in longitudinal section. Data represent means ± SE (*n* ≥ 30). (l) Statistical analysis of the number of pith cells per 4 × 10^4^ μm^2^ in longitudinal section. Data represent means ± SE (*n* = 15). Statistically significant differences compared to the control are marked with asterisks (*P < 0.05, **P < 0.01, ns indicates no significant difference, Student's *t*‐test).

To further investigate the function of *Ci‐miR156c* in citrus, its expression was suppressed by the STTM (short tandem target mimic) method (Tang *et al*., 2012). 12 *Ci‐miR156c*‐STTM transgenic trifoliate orange lines were generated. Phenotypic observations showed that the plant height of the *Ci‐miR156c*‐STTM lines increased slightly compared to that of the control plants, whereas there was no difference in thorn length or leaf area (Figure 3a–d). Subsequently, two transgenic lines (#2 and #6) from the *Ci‐miR156c*‐STTM transgenic trifoliate orange were randomly selected for phenotypic analysis. The expression levels of *Ci‐MIR156c* and *Ci‐miR156* were significantly reduced by 50% in the two transgenic lines (Figure S5c,d). Further statistical analysis of plant height, leaf number, average internode length, thorn length, and leaf area revealed that the *Ci‐miR156c*‐STTM lines displayed increased plant height and internode length compared with the controls, whereas the leaf number, thorn length, and leaf area were not significantly different (Figure 3e–i). Paraffin section analysis of the longitudinal section of the internode showed that the pith cell diameter of the *Ci‐miR156c*‐STTM lines was significantly greater than that of the controls, and the number of pith cells was decreased (Figure 3j–l). These results indicate that *Ci‐miR156c* may negatively regulate the height of citrus plants. However, the *Ci‐miR156c*‐STTM transgenic trifoliate orange showed no difference in flowering phenotype compared to the controls.

### Transcriptome analysis of *Ci‐miR156c* transgenic citrus

**Figure 4:**
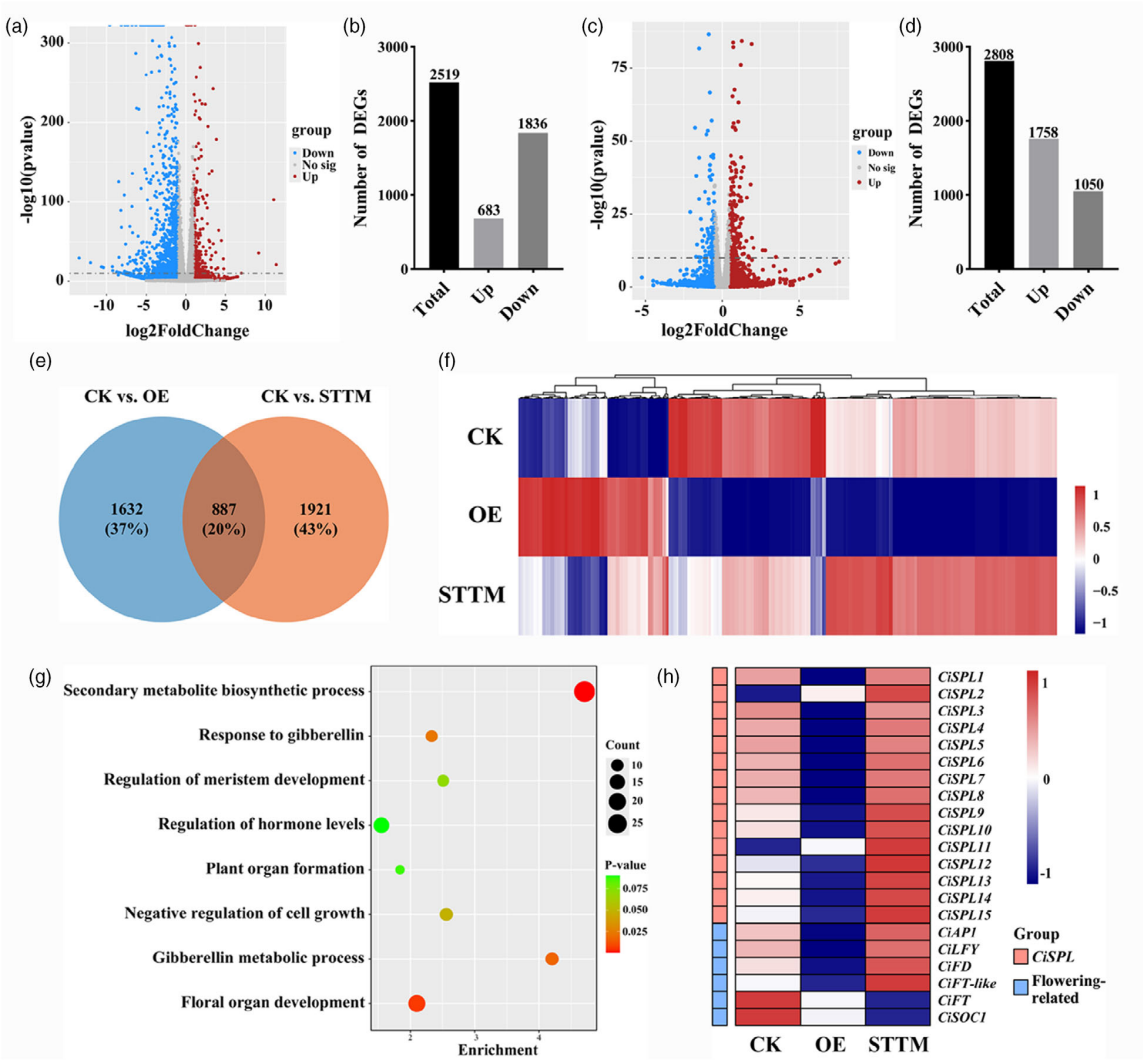
Transcriptome analysis of *Ci‐miR156c* transgenic citrus. (a) Distribution of differentially expressed genes (DEGs) between *Ci‐miR156c*‐OE transgenic trifoliate orange and the control. (b) Total number of DEGs between *Ci‐miR156c*‐OE transgenic trifoliate orange and the control. (c) Distribution of DEGs between *Ci‐miR156c*‐STTM transgenic trifoliate orange and the control. (d) Total number of DEGs between *Ci‐miR156c*‐STTM transgenic trifoliate orange and the control. (e) Venn diagram of DEGs between the CK versus OE and CK versus STTM comparisons. CK represents the control. OE represents *Ci‐miR156c*‐OE transgenic trifoliate orange. STTM represents *Ci‐miR156c*‐STTM transgenic trifoliate orange. (f) Heatmap diagram of common DEGs between CK, OE, and STTM. Log_2_ values of fragments per kilobase million (FPKM) were used to generate the heatmap. Relative expression level is indicated on a colour scale from magenta (high) to blue (low). (g) Biological process analysis of common DEGs between CK, OE, and STTM by gene ontology analysis. (h) Expression profiles of *CiSPL* and flowering‐related genes in CK and *Ci‐miR156c* transgenic trifoliate orange. Log_2_ values of FPKM were used to generate the heat map. Relative expression level is indicated on a colour scale from magenta (high) to blue (low).

To identify downstream target genes, the shoot apex was collected from the control, *Ci‐miR156c*‐OE, and *Ci‐miR156c*‐STTM transgenic plants for RNA‐seq analysis. A total of 2519 genes (683 upregulated and 1836 downregulated) were differentially expressed between *Ci‐miR156c*‐OE and the control, and 2808 genes (1758 upregulated and 1050 downregulated) were differentially expressed between *Ci‐miR156c*‐STTM and the control (Figure 4a–d, Tables S1 and S2). Subsequently, gene ontology (GO) analysis revealed that the most enriched biological processes based on the differentially expressed genes (DEGs) between *Ci‐miR156c*‐OE and the control were related to the regulation of hormone levels, secondary metabolite biosynthetic process, GA‐mediated signalling pathway, floral organ development, negative regulation of developmental process, response to GA, and vegetative phase change (Figure S7a). Further GO analysis of DEGs between *Ci‐miR156c*‐STTM and the control showed that the most enriched biological processes were related to hormone metabolic process, floral organ development, regulation of signal transduction, regulation of meristem growth, GA‐mediated signalling pathway, and response to GA (Figure S7b).

Based on the Venn diagram analysis, 4440 DEGs were identified, of which 887 were common DEGs between the control versus *Ci‐miR156c*‐OE and control versus *Ci‐miR156c*‐STTM comparisons (Figure 4e). To better understand the expression trends, hierarchical clustering was performed and a heatmap revealed that the DEGs were clustered into upregulated or downregulated groups (Figure 4f). The results indicated that more genes were downregulated in *Ci‐miR156c*‐OE and upregulated in *Ci‐miR156c*‐STTM compared to the control. Gene ontology (GO) analysis of the 887 common DEGs showed that the most enriched biological processes included secondary metabolite biosynthetic process, floral organ development, regulation of hormone levels, GA metabolic process, negative regulation of cell growth, and regulation of meristem growth (Figure 4g). These results suggested that *Ci‐miR156c* might play an important role in plant development and hormone‐related processes. Due to the conservation of *miR156*‐*SPL* regulatory module in different plants (Feng *et al*., 2023; Liu *et al*., 2017a; Xing *et al*., 2010), all DEGs were analysed, and it was found that most *CiSPL* genes were downregulated in *Ci‐miR156c*‐OE plants and upregulated in *Ci‐miR156c*‐STTM plants compared with the control (Figure 4h). Further analysis of the RNA‐seq results showed that some flowering‐related genes, such as *CiFT*, *CiLFY*, *CiAP1*, and *CiFD* were downregulated in *Ci‐miR156c*‐OE and upregulated in *Ci‐miR156c*‐STTM lines compared with the controls (Figure 4h).

### *Ci‐miR156c* regulates flowering by targeting *CiFD* in citrus

**Figure 5:**
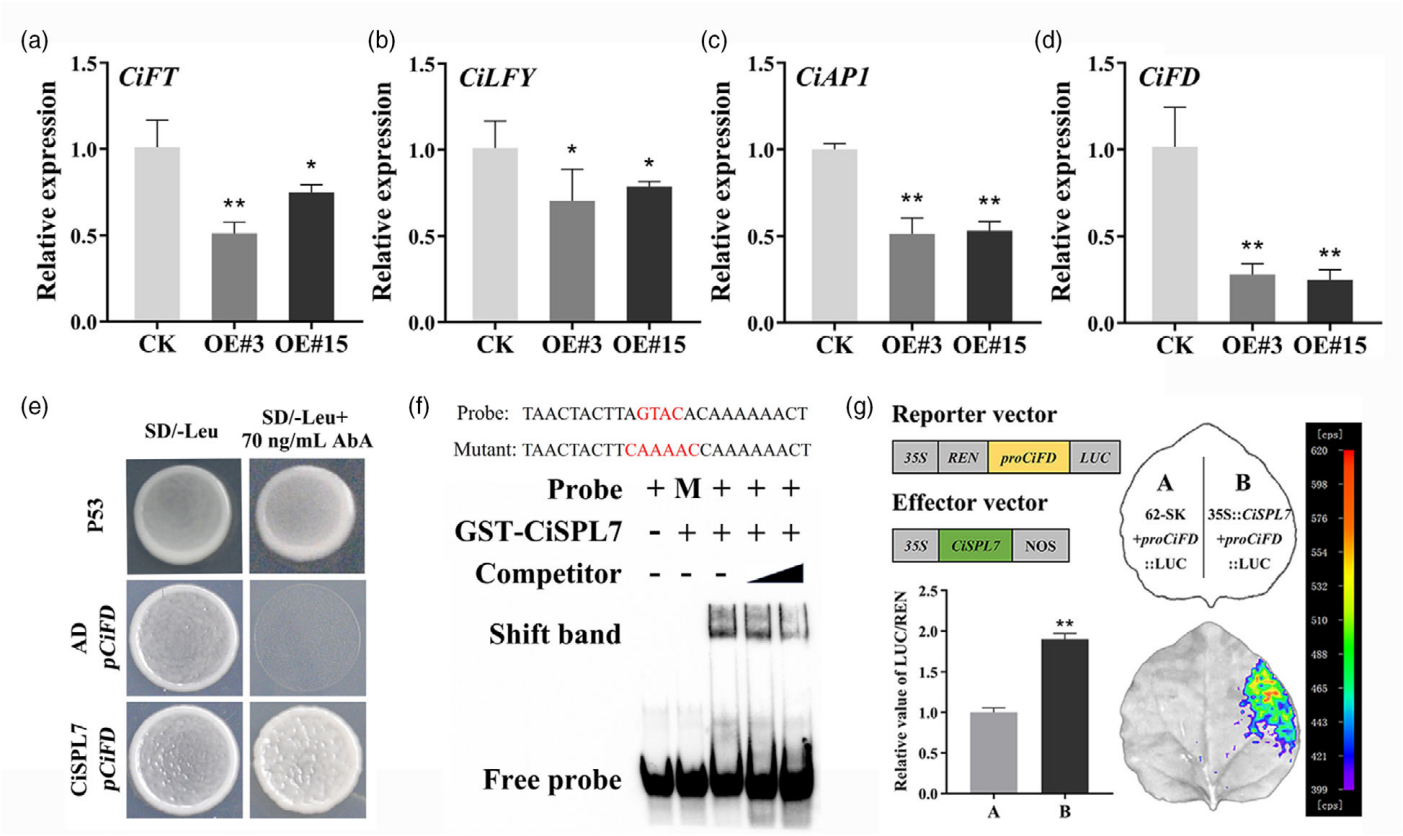
*Ci‐miR156c*‐*CiSPL* module regulates citrus flowering by targeting *CiFD*. (a–d) The expression analysis of *CiFT*, *CiLFY*, *CiAP1*, and *CiFD* in the shoot apex of 2‐year‐old *Ci‐miR156c* transgenic trifoliate orange. Citrus *Actin* was used as the internal reference gene, and CK was used as the control (with relative expression level set as 1.0). (e) Yeast one‐hybrid assay confirmed the interaction of CiSPL7 and the *CiFD* promoter. Yeast cells co‐transformed with CiSPL7 and the *CiFD* promoter grew well on SD/‐Leu plates or SD/‐Leu plates supplemented with 70 ng/mL AbA. P53 was used as the positive control, AD + *pCiFD* was used as the negative control. (f) EMSA confirmed that GST‐CiSPL7 binds to the GTAC *cis*‐element in the *CiFD* promoter. The shift band indicates the position of the protein‐DNA complex after incubation of the biotin‐labelled DNA probe and the GST‐CiSPL7 protein. + and – represent the presence (+) or absence (−) of the components. M represents the mutant probe. The black triangle represents an increase in the proportion of unlabelled competing probe from 2.5‐fold to 10‐fold compared with biotin‐labelled probe. (g) LUC activity measurement in *Nicotiana benthamiana* leaves after co‐expression of *35S*::CiSPL7 and *pCiFD*::Luc. The combination of empty vector (62‐SK) and *pCiFD*::Luc was used as the control. Data represent means ± SE (*n* = 3). Statistically significant differences compared to the control are marked with asterisks (*P < 0.05, **P < 0.01, ns indicates no significant difference, Student's *t*‐test).

*miR156* is a conserved miRNA family that targets and inhibits *SPL* genes. These genes encode proteins containing a conserved SBP domain, which binds to the GTAC *cis*‐element to regulate downstream gene expression (Axtell and Bowman, 2008; Yang *et al*., 2022). In citrus, previous studies have shown that *Ci‐miR156* targets nine *CiSPL* genes with the intact SBP domain (Liu *et al*., 2017b; Wu *et al*., 2010, 2015). Consistent with this targeting relationship, RT‐qPCR analysis showed that the expression of these *SPL* genes was significantly suppressed in *Ci‐miR156c*‐OE lines and upregulated in *Ci‐miR156c*‐STTM lines compared to that in the controls (Figure S8). Consistent with the RNA‐seq results, RT‐qPCR also showed that *CiFT*, *CiLFY*, *CiAP1*, and *CiFD* were significantly downregulated in *Ci‐miR156c*‐OE lines compared to the controls (Figure 5a–d). Previous studies have demonstrated that overexpression of *CiFT* and *CiFD* promotes flowering in citrus (Wu *et al*., 2022; Ye *et al*., 2023).

To further investigate whether *CiFT* and *CiFD* function as downstream regulatory genes of *Ci‐miR156c*, their promoter regions were cloned and analysed for potential SPL‐binding sites using promoter element analysis (Figure S9a,b). Yeast one‐hybrid (Y1H) assay showed that CiSPL7 could bind to the *CiFD* promoter, whereas the *Ci‐miR156c*‐*CiSPL* module exhibited no direct interaction with the *CiFT* promoter (Figure 5e and Figure S9c,d). Moreover, electrophoretic mobility shift assay (EMSA) demonstrated the interaction of CiSPL7 with the *CiFD* promoter (Figure 5f). Subcellular localization demonstrated that CiSPL7 was localized in the nucleus, and a transcriptional activity assay showed that CiSPL7 functioned as a transcriptional activator (Figure S10). The dual‐luciferase assay was performed in the epidermal cells of tobacco leaves. Compared to the controls, the ratio of LUC/REN was significantly increased upon co‐transformation with *35S*::CiSPL7 + *proCiFD*::LUC. It suggested that CiSPL7 positively regulates the transcription of *CiFD* (Figure 5g). These results indicated that *Ci‐miR156c* is involved in the flowering regulation of citrus by inhibiting *CiSPL7*, which further activates *CiFD*.

In *Arabidopsis*, FD directly activates the *SPL3/4/5* genes, indicating that the *FT‐FD* module regulates *SPL* genes in the shoot apex to control flowering time (Jung *et al*., 2016). FD binds to the C‐box (GACGTC) or G‐box (CACGTG) elements in the promoters of its target genes (Jung *et al*., 2012). To investigate whether a similar *FD*‐mediated regulatory mechanism exists in citrus, we first analysed the promoter sequences of 15 *CiSPL* genes for potential FD binding sites (C‐box/G‐box). Among these, only *CiSPL3*, *CiSPL9*, and *CiSPL11* promoters contained these motifs (Figure S11a). We then performed yeast one‐hybrid assays (Y1H) to test whether CiFD binds to these three candidate promoters. The assays confirmed that CiFD binds to the *CiSPL3* promoter (Figure S11b). However, no interaction was detected between CiFD and the promoters of *CiSPL9* or *CiSPL11* (Figure S11b).

### *Ci‐miR156c*‐ *CiSPL* module regulates *CiKN6* expression

**Figure 6:**
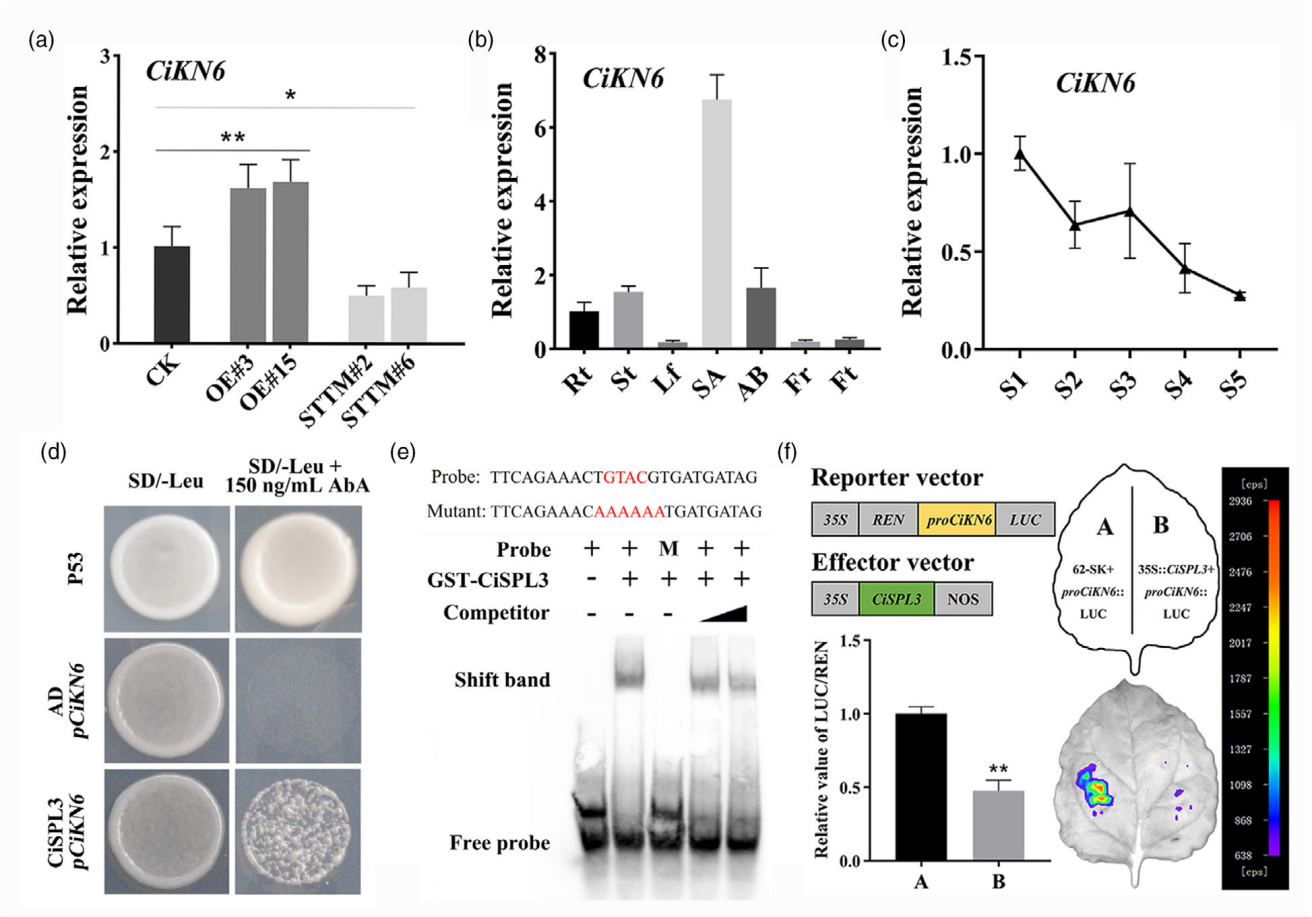
*Ci‐miR156c*‐*CiSPL* module regulates citrus architecture by targeting *CiKN6*. (a) The expression analysis of *CiKN6* in the shoot apex of 6‐month‐old *Ci‐miR156c* transgenic trifoliate orange. CK represents the control. OE#3 and OE#15 represent two *Ci‐miR156c‐*OE transgenic lines. STTM#2 and STTM#6 represent two *Ci‐miR156c*‐STTM transgenic lines. CK was used as the control (with relative expression level set as 1.0). (b) The expression of *CiKN6* in various tissues of adult trifoliate orange. AB, Axillary bud from spring shoot; Fr, Flower at full bloom; Ft, Fruit (young fruit from 30 days after flowering); Lf, Healthy mature leaf; Rt, Lateral root; SA, Shoot apex from spring shoot; St, Stem from spring shoot. Rt was used as the control (with relative expression level set as 1.0). (c) The expression level of *CiKN6* during plant height development. S: Stage, S1–S5 represent different development stages with increasing plant height. S1 was used as the control (with relative expression level set as 1.0). Citrus *Actin* was used as the internal reference gene. (d) Yeast one‐hybrid assay confirmed the interaction of CiSPL3 and the *CiKN6* promoter. Yeast cells co‐transformed with CiSPL3 and the *CiKN6* promoter grew well on SD/‐Leu plates or SD/‐Leu plates supplemented with 150 ng/mL AbA. P53 was used as the positive control, AD + *pCiKN6* was used as the negative control. (e) EMSA confirmed that GST‐CiSPL3 binds to the GTAC *cis*‐element in the *CiKN6* promoter. The shift band indicates the position of the protein–DNA complex after incubation of the biotin‐labelled DNA probe and the GST‐CiSPL3 protein. + and – represent presence (+) or absence (−) of the components. M represents the mutant probe. The black triangle represents an increase in the proportion of unlabeled competing probe from 2.5‐fold to 10‐fold compared with biotin‐labelled probe. (f) LUC activity measurement in *Nicotiana benthamiana* leaves after co‐expression of *35S*::CiSPL3 and *pCiKN6*::Luc. The combination of empty vector (62‐SK) and *pCiKN6*::Luc was used as the control. Data represent means ± SE (*n* = 3). Statistically significant differences compared with the control are marked with asterisks (*P < 0.05, **P < 0.01, ns indicates no significant difference, Student's *t*‐test).

**Figure 7:**
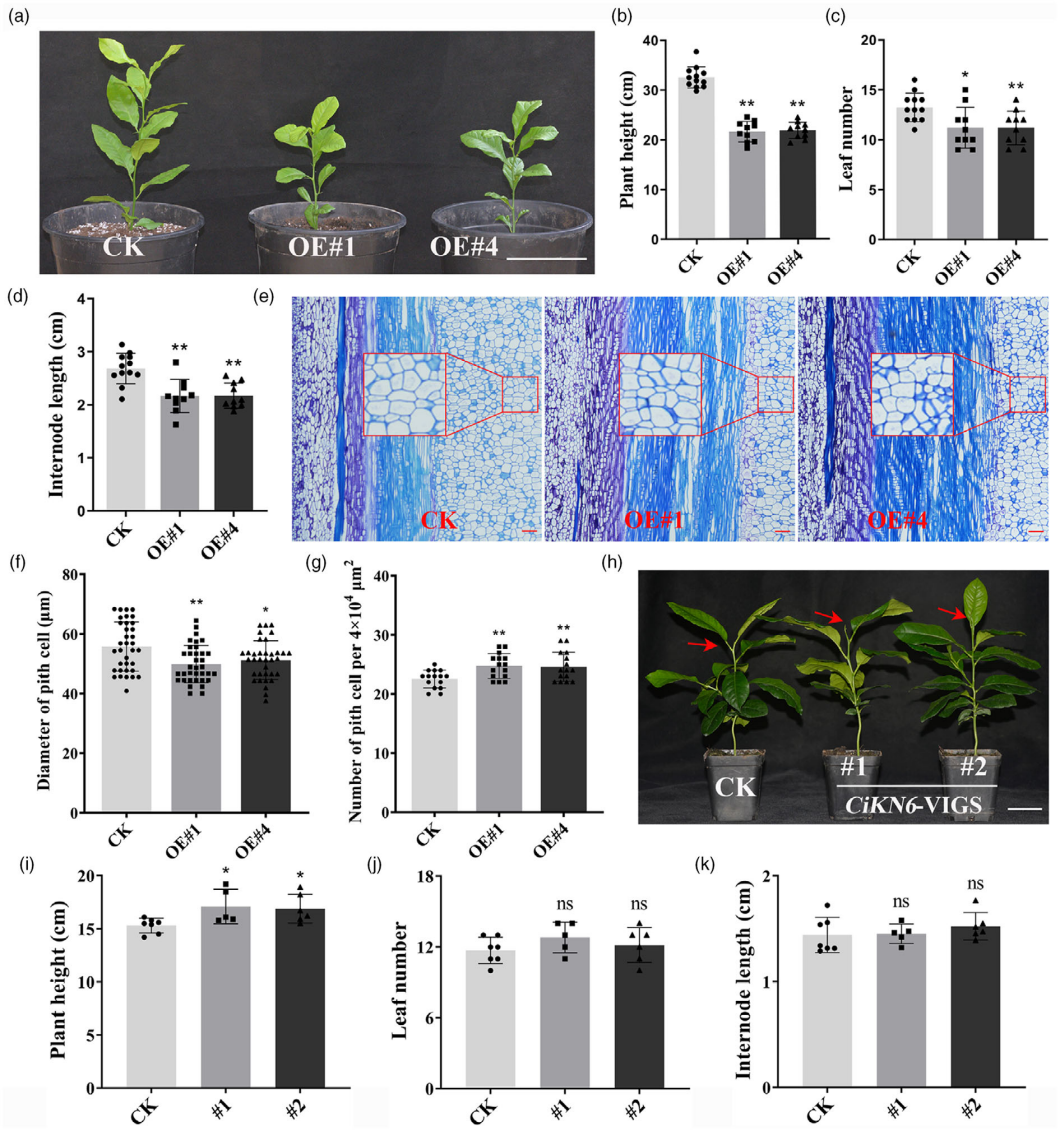
Phenotype analysis of *CiKN6*‐OE (overexpression) transgenic citrus. (a) Phenotypic characteristics of 5‐month‐old *CiKN6*‐OE transgenic lemon. CK represents the control. OE#1 and OE#4 represent two *CiKN6*‐OE transgenic lines. Scale bar = 15 cm. (b–d) Statistical analysis of plant height (b), leaf number (c), and internode length (d) in *CiKN6*‐OE transgenic lemon compared with the control. Data represent means ± SE (*n* = 12). (e) Longitudinal section of the 5th internode (from top to bottom) of 5‐month‐old control and *CiKN6*‐OE transgenic lemon. Scale bar = 100 μm. The red boxes indicate randomly selected areas for subsequent statistical analysis of the diameter of pith cells and the number of pith cells. (f) Statistical analysis of the diameter of pith cells in the longitudinal section. Data represent means ± SE (*n* ≥ 30). (g) Statistical analysis of the number of pith cells per 4 × 10^4^ μm^2^ in the longitudinal section. Data represent means ± SE (*n* = 15). (h) Phenotypic characteristics of 3‐month‐old *CiKN6*‐VIGS transgenic lemon. CK represents the control. #1 and #2 represent two *CiKN6*‐VIGS transgenic lines. The red arrow represents the shoot apex of plants. Scale bar = 5 cm. (i–k) Statistical analysis of plant height (i), leaf number (j), and internode length (k) in *CiKN6*‐VIGS transgenic lemon compared with the control. Data represent means ± SE (*n* ≥ 5). Statistically significant differences compared to the control are marked with asterisks (*P < 0.05, **P < 0.01, ns indicates no significant difference, Student's *t*‐test).

Previous studies have indicated that *KNOX* genes play a central role in SAM maintenance and that SAM development is closely related to plant architecture (Su *et al*., 2020; Tsuda *et al*., 2011). Among the DEGs in the RNA‐seq results, 12 citrus *KNOX* homologous genes were found (Figure S12a). Several *KNOX* genes were significantly upregulated in *Ci‐miR156c*‐OE plants and downregulated in *Ci‐miR156c*‐STTM plants, indicating that these genes might be involved in regulating plant height in citrus. To further investigate this possibility, *cis*‐elements were analysed in the 2000‐bp promoter regions of these *CiKNOX* genes, revealing that all contain the SPL binding site (Figure S12b). Among these *KNOX* genes, *CiKN6* (Ciclev10001779m) was found to be associated with citrus leaf development in a previous study (Zeng *et al*., 2022) and dwarfism compared with the controls (Figure 7a). Based on these findings, it was hypothesized that the *Ci‐miR156c*‐*CiSPL* module may regulate citrus plant height by targeting *CiKN6*. Analysis of *CiKN6* expression revealed that it was significantly upregulated in *Ci‐miR156c*‐OE lines and downregulated in *Ci‐miR156c*‐STTM lines compared to the controls (Figure 6a). Spatial expression analysis showed that *CiKN6* was highly expressed in the shoot apex, stem, and axillary bud (Figure 6b). RT‐qPCR showed that the expression of *CiKN6* was also decreased with increasing plant height (Figure 6c).

To investigate whether the *Ci‐miR156c*‐*CiSPL* module regulates the expression of *CiKN6*, we first cloned the promoter fragment of *CiKN6* containing the SPL binding site (Figure S13a). Subsequently, a Y1H assay was performed, which confirmed that CiSPL3 binds to the *CiKN6* promoter (Figure 6d and Figure S13b). The interaction between CiSPL3 and *CiKN6* was verified using EMSA (Figure 6e). Subcellular localization demonstrated that CiSPL3 was localized in the nucleus, and transcriptional activity analysis showed that CiSPL3 lacks transcriptional activation activity (Figure S14). Furthermore, the dual‐luciferase assay showed that CiSPL3 significantly repressed the transcription of *CiKN6* (Figure 6f). These results suggested that *Ci‐miR156c* regulates citrus plant height by inhibiting *CiSPL3*, which further represses *CiKN6*.

### *CiKN6* participates in plant height development

To further explore the function of *CiKN6* in citrus, 11 *CiKN6*‐OE transgenic lemon lines were obtained in a previous study (Zeng *et al*., 2022). Two transgenic lines (#1 and #4) from the *CiKN6*‐OE transgenic lemons were randomly selected for phenotypic analysis (Figure 7a). The expression levels of *CiKN6* in the two transgenic lines were higher than those in the controls (Figure S15a). Further statistical analysis showed that plant height, leaf number, and average internode length of the *CiKN6*‐OE lines were significantly reduced compared with those of the controls (Figure 7b–d). Paraffin section analysis of the longitudinal section of internode showed that the pith cell diameter in the *CiKN6*‐OE lines was significantly smaller than that in the controls, and the number of pith cells was increased (Figure 7e–g). Virus‐induced gene silencing (VIGS) technology was used to suppress the expression of *CiKN6* in lemon. The expression of *CiKN6* was significantly suppressed in *CiKN6*‐VIGS plants compared to that in the controls (Figure S15b). After 3 months of growth and development under normal conditions, the plant height of *CiKN6*‐VIGS plants was significantly higher than that of the controls, whereas leaf number and average internode length showed no significant difference from those of the controls (Figure 7h–k). These results indicated that *CiKN6* may be involved in the regulation of plant height in citrus.

### Effects of altered expression of *Ci‐miR156c* and *CiKN6* on endogenous GA content

**Figure 8:**
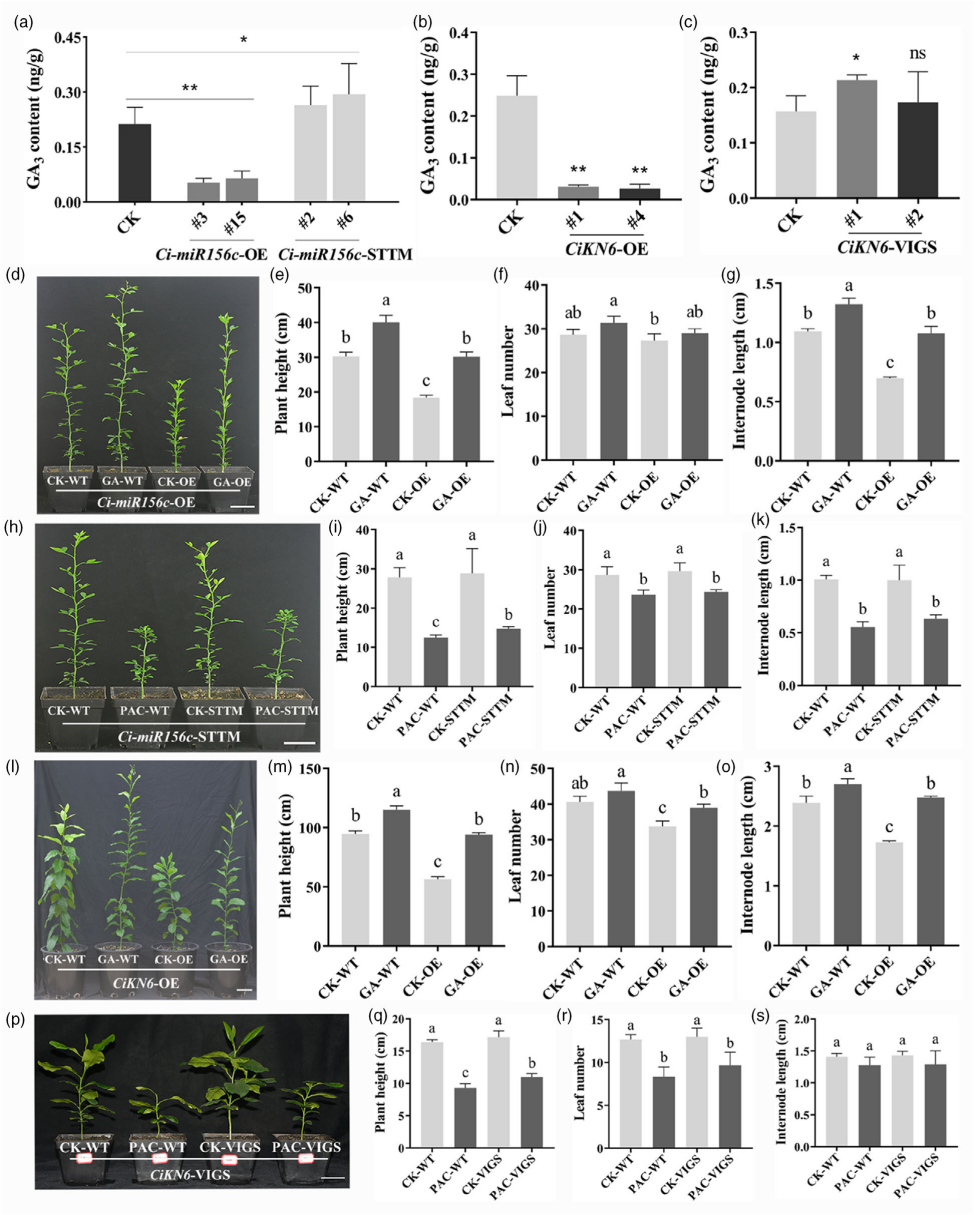
Phenotypic analysis of transgenic plants with GA_3_ and PAC treatments. (a) GA_3_ content in the shoot apex (including some new leaves) of 6‐month‐old control, *Ci‐miR156c*‐OE, and *Ci‐miR156c*‐STTM transgenic trifoliate orange. CK represents the control. #3 and #15 represent two *Ci‐miR156c‐*OE transgenic lines. #2 and #6 represent two *Ci‐miR156c*‐STTM transgenic lines. (b, c) GA_3_ content in the shoot apex (including some new leaves) of 5‐month‐old *CiKN6*‐OE (b) and 3‐month‐old *CiKN6*‐VIGS (c) transgenic lemon. CK represents the control. #1 and #4 represent two *CiKN6*‐OE transgenic lines. #1 and #2 represent two *CiKN6*‐VIGS transgenic lines. Data represent means ± SE (*n* = 3). Statistically significant differences compared to the control is marked with asterisk(s) (*P < 0.05, **P < 0.01, ns indicates no significant difference, Student's *t*‐test). (d) Phenotypic analysis of *Ci‐miR156c*‐OE transgenic trifoliate orange after GA_3_ treatment. Scale bar = 5 cm. (e–g) Statistical analysis of plant height (e), leaf number (f), and internode length (g) after GA_3_ treatment. (h) Phenotypic analysis of *Ci‐miR156c*‐STTM transgenic trifoliate orange after PAC treatment. Scale bar = 5 cm. (i–k) Statistical analysis of plant height (i), leaf number (j), and internode length (k) after PAC treatment. (l) Phenotypic analysis of *CiKN6*‐OE transgenic lemon after GA_3_ treatment. Scale bar = 10 cm. (m–o) Statistical analysis of plant height (m), leaf number (*n*), and internode length (o) after GA_3_ treatment. (p) Phenotypic analysis of *CiKN6*‐VIGS transgenic lemon after PAC treatment. Scale bar = 5 cm. (q–s) Statistical analysis of plant height (q), leaf number (r), and internode length (s) after PAC treatment. Data represent means ± SE (*n* = 3). Different letters above the bars indicate a significant difference according to Tukey's multiple range test (*P* < 0.05).

GA regulates plant height by promoting cell division and elongation, and their decisive role in plant height regulation has been demonstrated in citrus (Fagoaga *et al*., 2007). RNA‐seq results revealed that DEGs between *Ci‐miR156c* transgenic plants and controls were enriched in the regulation of hormone levels, response to GA, and GA‐mediated signalling pathway (Figure 4g and Figure S7). To verify whether the dwarfing phenotypes of *Ci‐miR156c*‐OE and *CiKN6*‐OE transgenic plants were associated with altered GA content, the endogenous GA content of the controls and transgenic plants was determined. The results showed that GA_3_ decreased significantly in *Ci‐miR156c*‐OE and increased significantly in *Ci‐miR156c*‐STTM transgenic citrus compared with that in the controls (Figure 8a). Furthermore, the content of GA_3_ was also decreased in *CiKN6*‐OE plants and slightly increased in *CiKN6*‐VIGS plants compared with that in the controls (Figure 8b,c). However, GA_1_, GA_4_, and GA_7_ were not detected in these transgenic plants. These results suggested that altered *Ci‐miR156c* and *CiKN6* expression may affect the endogenous GA_3_ content.

To analyse the role of GA_3_ in plant height regulation, transgenic plants were treated with exogenous GA_3_ and paclobutrazol (PAC, an GA inhibitor). Approximately, 3‐month‐old *Ci‐miR156c*‐OE plants were treated with 20 mg/L GA_3_, whereas *Ci‐miR156c*‐STTM plants were treated with 300 mg/L PAC. After 5 weeks of GA_3_ and PAC treatment, plant height, leaf number, and average internode length were measured. The GA_3_ treatment restored plant height, leaf number, and internode length in *Ci‐miR156c*‐OE lines to WT levels (Figure 8d–g). In contrast, PAC treatment reduced the height of *Ci‐miR156c*‐STTM plants compared with that of untreated STTM plants (Figure 8h,i). Furthermore, PAC‐treated *Ci‐miR156c*‐STTM plants showed a decrease in leaf number and average internode length compared with untreated STTM plants (Figure 8j,k).

Approximately, 5‐month‐old *CiKN6*‐OE plants were treated with 50 mg/L GA_3_, and 2‐month‐old *CiKN6*‐VIGS plants were treated with 300 mg/L PAC. After 5 weeks, the GA_3_ treatment restored plant height, leaf number, and internode length in *CiKN6*‐OE lines to WT levels (Figure 8l–o). In contrast, PAC treatment reduced the height of *CiKN6*‐VIGS plants compared with that of untreated VIGS plants (Figure 8p,q). Additionally, PAC‐treated *CiKN6*‐VIGS plants showed a decrease in leaf number and average internode length compared to untreated VIGS plants (Figure 8r,s). Taken together, GA_3_ treatment fully rescued the dwarfing phenotype of *Ci‐miR156c*‐OE and *CiKN6*‐OE plants, suggesting that the plant height phenotypes observed in the transgenic plants is likely due to altered GA_3_ levels.

### *Ci‐miR156c*‐ *CiSPL* module inhibits GA biosynthesis by binding to the *CiGA20ox2* promoter

**Figure 9:**
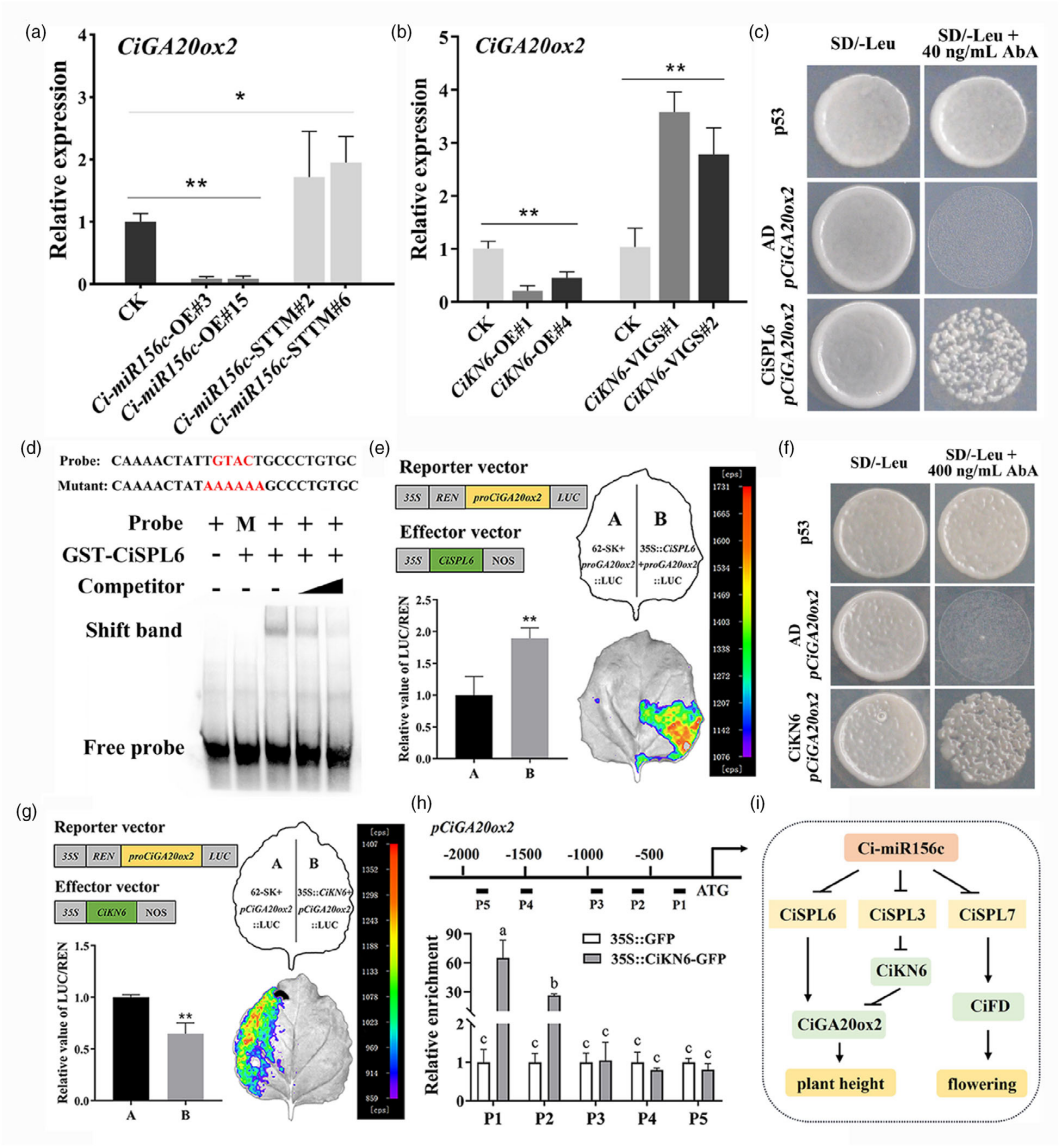
The *Ci‐miR156c*‐*CiSPL* module inhibits GA biosynthesis by binding to the *CiGA20ox2* promoter. (a) The expression analysis of *CiGA20ox2* in the shoot apex of 6‐month‐old *Ci‐miR156c*‐OE and *Ci‐miR156c*‐STTM transgenic trifoliate orange. CK represents the control. OE#3 and OE#15 represent two *Ci‐miR156c‐*OE transgenic lines. STTM#2 and STTM#6 represent two *Ci‐miR156c*‐STTM transgenic lines. (b) The expression analysis of *CiGA20ox2* in the shoot apex of 5‐month‐old *CiKN6*‐OE and 3‐month‐old *CiKN6*‐VIGS transgenic lemon. CK represents the control. OE#1 and OE#4 represent two *CiKN6*‐OE transgenic lines, and VIGS#1 and VIGS#2 represent two *CiKN6*‐VIGS transgenic lines. Citrus *Actin* was used as the internal reference gene, and CK was used as the control (with relative expression level set as 1.0). (c) Yeast one‐hybrid assay confirmed the interaction between CiSPL6 and the *CiGA20ox2* promoter. Yeast cells co‐transformed with CiSPL6 and the *CiGA20ox2* promoter grew well on SD/‐Leu plates or SD/‐Leu plates supplemented with 40 ng/mL AbA. P53 was used as the positive control, and AD + *pCiGA20ox2* was used as the negative control. (d) EMSA confirmed that GST‐CiSPL6 binds to the GTAC *cis*‐element in the *CiGA20ox2* promoter. The shift band indicates the position of the protein‐DNA complex after incubation of the biotin‐labelled DNA probe with the GST‐CiSPL6 protein. + and – represent presence (+) or absence (−) of the components. M represents the mutant probe. The black triangle represents an increase in the proportion of unlabeled competing probe from 2.5‐fold to 10‐fold compared to biotin‐labelled probe. (e) LUC activity measurement in *Nicotiana benthamiana* leaves after co‐expression of *35S*::CiSPL6 and *pCiGA20ox2*::Luc. The combination of empty vector (62‐SK) and *pCiGA20ox2*::Luc was used as the control. (f) Yeast one‐hybrid assay confirmed the interaction between CiKN6 and the *CiGA20ox2* promoter. Yeast cells co‐transformed with CiKN6 and the *CiGA20ox2* promoter grew well on SD/‐Leu plates or SD/‐Leu plates supplemented with 400 ng/mL AbA. P53 was used as the positive control, and AD + *pCiGA20ox2* was used as the negative control. (g) LUC activity was measured in *Nicotiana benthamiana* leaves after co‐expression of *35S*::CiKN6 and *pCiGA20ox2*::Luc. The combination of empty vector (62‐SK) and *pCiGA20ox2*::Luc was used as the control. Data represent means ± SE (*n* = 3). Statistically significant differences to the control are marked with asterisk(s) (*P < 0.05, **P < 0.01, ns indicates no significant difference, Student's *t*‐test). (h) CiKN6 enriched in the P1 (from −195 to −313 bp) and P2 (from −531 to −633 bp) fragments of *CiGA20ox2* promoter by ChIP‐qPCR. P3, P4, and P5 fragments as the negative control. *35S*::GFP transgenic line was used as empty control. Data represent means ± SE (*n* = 3). Different letters above the bars indicate a significant difference according to Tukey's multiple range test (*P* < 0.05). (i) A working model of *Ci‐miR156c* regulation of citrus plant height and flowering.

Previous studies have confirmed that overexpression of *CiGA20ox2* increased plant height in citrus (Fagoaga *et al*., 2007; Kotoda *et al*., 2016; Vidal *et al*., 2001). The expression of *CiGA20ox2* was upregulated with increasing plant height (Figure S16). Expression was also investigated in *Ci‐miR156c* and *CiKN6* transgenic plants. The results showed that *CiGA20ox2* was downregulated in *Ci‐miR156c*‐OE and *CiKN6*‐OE plants and upregulated in *Ci‐miR156c*‐STTM and *CiKN6*‐VIGS plants compared with the controls (Figure 9a,b). Based on the altered GA_3_ content and the expression of *CiGA20ox2* in these transgenic plants, we speculated that *Ci‐miR156c*‐*CiSPL* might directly regulate *CiGA20ox2* or indirectly target it through the *Ci‐miR156c*‐*CiSPL*‐*CiKN6* pathway. To verify this hypothesis, the *CiGA20ox2* promoter was cloned and a potential SPL‐binding site was identified (Figure S17a). Subsequently, the Y1H assay demonstrated that CiSPL6 bound to the promoter of *CiGA20ox2* (Figure 9c and Figure S17b). EMSA further supported the direct interaction between CiSPL6 and *CiGA20ox2* (Figure 9d). Subcellular localization confirmed that CiSPL6 was localized in the nucleus, and transcriptional activity assays indicated that CiSPL6 likely functioned as a transcriptional activator (Figure S18). Dual‐luciferase assays revealed that CiSPL6 significantly promoted the transcription of *CiGA20ox2* (Figure 9e). Regarding the regulation of *CiSPLs*, previous studies using RNA ligase‐mediated rapid amplification of cDNA ends (RLM‐RACE) and degradome sequencing confirmed that *Ci‐miR156* directs the cleavage of *CiSPL3*/*6*/*7* in citrus (Wu *et al*., 2010, 2015). Building on these findings, this study further validated the *in vivo* interaction between *Ci‐miR156c* and *CiSPL3/6/7* through transient expression assays (Figure S19).

Previous studies confirmed that KNOX proteins bind to TGAC *cis*‐element and regulate the expression of downstream genes (Bolduc *et al*., 2012; Zhao *et al*., 2020). Analysis of *CiGA20ox2* promoter revealed the presence of potential KNOX‐binding *cis*‐element. The Y1H assay demonstrated that CiKN6 binds to the *CiGA20ox2* promoter (Figure 9f). A previous study showed that CiKN6 is localized in the nucleus and may function as a transcriptional suppressor (Zeng *et al*., 2022). The dual‐luciferase assay indicated that CiKN6 significantly repressed the expression of *CiGA20ox2* (Figure 9g). To validate this interaction *in vivo*, *35S*::GFP and *35S*::*CiKN6*‐GFP transgenic citrus plants were used in a chromatin immunoprecipitation (ChIP) assay. ChIP‐qPCR results confirmed that the promoter of *CiGA20ox2* was significantly enriched in the P1 and P2 fragments using antibodies recognizing the CiKN6‐GFP protein, but not in the P3, P4, or P5 fragments (Figure 9h). The P1 and P2 fragments contain KNOX‐binding sites. Therefore, the *Ci‐miR156c*‐*CiSPL* module might directly bind to the *CiGA20ox2* promoter or indirectly regulate it through the *Ci‐miR156c*‐*CiSPL3*‐*CiKN6* pathway, thereby inhibiting GA biosynthesis in *Ci‐miR156c*‐OE and *CiKN6*‐OE transgenic plants.

## Discussion

Flowering represents a critical transition from vegetative to reproductive growth in plants (Bao *et al*., 2020). However, conventional breeding and genetic improvement processes of woody plants are significantly constrained by their prolonged juvenile phase (Agustí *et al*., 2022). Therefore, shortening the juvenile period is a key objective in the breeding of perennial woody plants. Plant architecture emerges through complex interactions between multiple factors, including overall stature, branching architecture, and growth periodicity (Hollender and Dardick, 2015; Wang and Li, 2008). An ideal tree structure is particularly important for increasing the yield and reducing management costs of woody plants, especially fruit crops (Hollender and Dardick, 2015). Consequently, the molecular mechanisms underlying plant architecture and flowering regulation have been extensively studied in woody plants, including citrus (Chu *et al*., 2023), peach (Cheng *et al*., 2019), poplar (Song *et al*., 2021), and apple (Feng *et al*., 2023). Recent studies on citrus have reported that a key gene for *ACC synthase* (*ACS4*) can regulate not only plant height but also flowering (Chu *et al*., 2023, 2024). However, the regulatory networks of plant height and flowering in woody plants remain unclear. In this study, *Ci‐miR156b* and *Ci‐miR156c* overexpression in tobacco caused dwarfism, with *Ci‐miR156c* having a stronger effect. Since *Ci‐MIR156c* expression was higher than *Ci‐MIR156b* in citrus, it was selected for further study. Overexpression of *Ci‐miR156c* in citrus delayed flowering and altered plant height. Considering the similarity between the phenotypes induced by *Ci‐miR156b* and *Ci‐miR156c* when ectopically expressed in tobacco, combined with the conservation of the *Ci‐miR156–CiSPL* regulatory module, it remains to be explored whether *Ci‐miR156b* regulates flowering and plant height in citrus, which also provides valuable clues for analysing these traits.

*miR156*, which is highly conserved in plants, regulates vegetative–reproductive phase transition through temporal or spatial accumulation (Xie *et al*., 2020). Overexpression of *miR156* delays flowering in *Arabidopsis*, rice, potato, switchgrass, and alfalfa (Aung *et al*., 2015; Bhogale *et al*., 2014; Fu *et al*., 2012; Mathieu *et al*., 2009; Wang *et al*., 2009). Similar effects have been observed in perennial woody plants such as Chinese Chestnut and Chinese fir (Chen *et al*., 2019). In the present study, the overexpression of *Ci‐miR156c* in tobacco and citrus resulted in late flowering, indicating that *miR156* is evolutionarily conserved in phase transition regulation across both annual herbaceous and perennial woody plants. Increasing evidence indicates that *miR156* targets *SPL* transcription factors, thereby forming a crucial regulatory module in plant development (Axtell and Bowman, 2008). *miR156*‐targeted *SPL* genes regulate key transcription factors: *SPL3/4/5* induce *LFY*, *AP1*, and *FRUITFULL* (*FUL*) to promote flowering (Yamaguchi *et al*., 2009), whereas *SPL9* promotes flowering by upregulating the expression of *SUPPRESSOR OF OVEREXPRESSION OF CONSTANS1* (*SOC1*), *FUL*, and *AGAMOUS LIKE 42* (*AGL42*) (Wang *et al*., 2009). In addition, the *miR156*–*SPL9* module controls the flowering time by regulating *miR172* (Wu *et al*., 2009), which targets *APETALA2* (*AP2*)‐related factors that suppress *FT* expression (Mathieu *et al*., 2009; Wang and Wang, 2015). The existence of such a regulatory mechanism could explain the downregulation of *CiFT* expression in *Ci‐miR156c* overexpressing plants in this study, but not through direct targeting of the *Ci‐miR156c–CiSPL* module. In *Arabidopsis*, FD directly activates the *SPL3/4/5* genes in the shoot apex to control flowering time (Jung *et al*., 2016). Here, we demonstrate a similar mechanism in citrus, where CiFD directly binds to the *CiSPL3* promoter. However, we also discovered a novel pathway in which *Ci‐miR156c* targets and represses *CiSPL7* expression, thereby preventing it from binding to the *CiFD* promoter to activate its expression. This mechanism has not been previously reported in model plants.

Plant height is an important agronomic and horticultural trait. In rice, *OsmiR156f* modulates plant architecture by repressing *OsSPL3/12/14* genes, a process that is likely mediated by a protease inhibitor I family protein (Liu *et al*., 2015). Conversely, *miR156ab* overexpression in apple resulted in greater leaf area and increased plant height (Feng *et al*., 2023). In this study, *Ci‐miR156c* negatively regulated plant development in trifoliate orange, leading to reduced plant height and leaf area. These results indicated that *miR156* exhibits functional variability during the development of different plants. However, *Ci‐miR156c*‐STTM plants displayed fewer phenotypic differences compared to the control than *Ci‐miR156c*‐OE transgenic plants, implying potential functional redundancy among miRNA family members. Additionally, the milder phenotypes observed in *Ci‐miR156c*‐STTM plants may also be attributed to their weaker effect on gene expression, with only a twofold decrease in expression compared to the fivefold or higher increase observed in the overexpression lines.

The *KNOX* family plays a crucial role in regulating plant height in various species, including citrus, poplar, and pear (Liu *et al*., 2022; Song *et al*., 2021; Zeng *et al*., 2022). For example, in citrus, overexpression of *CsKN1* prolongs vegetative growth in the SAM and alters leaf morphology (Zeng *et al*., 2021). In our study, several *CiKNOX* genes were mis‐regulated in the *Ci‐miR156c* transgenic lines. Among these, a previous study demonstrated that *CiSPL11*‐regulated *CiKN6* regulates citrus leaf development and is associated with a dwarfing phenotype (Zeng *et al*., 2022). This finding led us to hypothesize that the *Ci‐miR156c‐CiSPL* module may target *CiKN6* to regulate plant height. Consistent with this hypothesis, our results suggest that the *Ci‐miR156c‐CiSPL3* module influences citrus plant height by modulating *CiKN6* expression. Furthermore, we identified putative SPL binding sites in the promoters of other *CiKNOX* genes whose expression was also altered by *Ci‐miR156c*. Collectively, these findings suggest that the *Ci‐miR156c‐CiSPL* module may coordinately regulate multiple *KNOX* genes, potentially impacting not only plant height but also other developmental processes. However, the underlying regulatory mechanisms require further investigation.

We observed that *Ci‐miR156c* overexpressing plants had similar leaf numbers but significantly shorter internodes and smaller cell diameters compared to the controls, leading to a dwarf phenotype. This was linked to the reduced endogenous GA content, which restricted internode elongation. Further investigation revealed that the citrus *Ci‐miR156c*‐*CiSPL3*‐*CiKN6*‐*CiGA20ox2* and *Ci‐miR156c*‐*CiSPL6*‐*CiGA20ox2* modules directly affect plant height by regulating GA biosynthesis. *CiGA20ox2* has been demonstrated to be positively associated with plant height development in citrus (Fagoaga *et al*., 2007). Furthermore, our findings expanded the list of target genes regulated by the *Ci‐miR156c*‐*CiSPL* module in citrus, including *CiFD*, *CiKN6*, and *CiGA20ox2*. The *Ci‐miR156c*‐*CiSPL3*‐*CiKN6*‐*CiGA20ox2* and *Ci‐miR156c*‐*CiSPL6*‐*CiGA20ox2* modules have been implicated in the regulation of citrus plant height, whereas the *Ci‐miR156c*‐*CiSPL7*‐*CiFD* module was involved in flowering regulation. These regulatory processes involved different *CiSPL* genes, indicating that *CiSPL* genes are differentially regulated by *Ci‐miR156c* and that the *Ci‐miR156c*‐*CiSPLs* module serves as a regulatory hub for multiple traits during citrus growth and development. However, the specific roles of *CiSPL* genes require further investigation.

Based on these results, we proposed a model in which the *Ci‐miR156c*‐*CiSPL* module regulates citrus flowering and plant height (Figure 9i). In flowering regulation, *Ci‐miR156c* targets and represses the expression of *CiSPL7*, preventing it from binding to the *CiFD* promoter to activate its expression, thereby delaying flowering. In plant height regulation, *Ci‐miR156c* targets and inhibits the expression of *CiSPL3*, which binds to the *CiKN6* promoter and represses its expression. Subsequently, *CiKN6* inhibits the expression of the GA biosynthesis gene *CiGA20ox2* by binding to its promoter, ultimately reducing GA biosynthesis *in vivo*. Additionally, *Ci‐miR156c* represses the expression of *CiSPL6*, preventing it from binding to the *CiGA20ox2* promoter to activate its expression. This leads to a reduction in the GA content *in vivo*, resulting in plant dwarfism.

## Materials and methods

### Plant materials and growth conditions

For morphological analysis, transgenic plants including *Ci‐miR156c*‐OE and *Ci‐miR156c*‐STTM trifoliate orange, *CiKN6*‐OE lemon, and *CiKN6*‐VIGS lemon were cultivated in a greenhouse of Huazhong Agricultural University (Wuhan, China) under natural growth conditions. Plant height, internode number, leaf number, and average internode length were measured in 8‐month‐old *Ci‐miR156c*‐OE transgenic trifoliate orange, 6‐month‐old *Ci‐miR156c*‐STTM transgenic trifoliate orange, 5‐month‐old *CiKN6*‐OE transgenic lemon, and 3‐month‐old *CiKN6*‐VIGS transgenic lemon. *Ci‐miR156c*‐OE and *Ci‐miR156c*‐STTM transgenic trifoliate oranges were grown in 16 × 18 cm plastic pots, *CiKN6*‐OE transgenic lemons were grown in 30 × 30 cm pots, and *CiKN6*‐VIGS lemons were grown in 7 × 9 cm pots. All the pots were filled with a mixture of commercial soil and perlite at a ratio of 3:1.

For RT‐qPCR analysis, shoot apex and leaf samples were collected from the controls, transgenic trifoliate orange, and transgenic lemon, rapidly frozen in liquid nitrogen, and stored at −80 °C until further use. For the dual‐luciferase assay and subcellular localization analysis, tobacco (*Nicotiana benthamiana*) was grown in a growth chamber under controlled conditions with a 16 h light/8 h dark cycle at 25 °C.

### Plasmid construction and plant transformation

The precursor of *Ci‐miR156c* (Table S3) was cloned into the pBI121 vector using the ClonExpress One Step Cloning Kit (Vazyme, Nanjing, China). The *Ci‐miR156c*‐STTM vector was constructed using the STTM method. Briefly, a short tandem target mimic sequence (Table S4) designed to block the function of *Ci‐miR156c* was synthesized as previously described (Tang *et al*., 2012) and cloned into the pBI121 vector. The coding sequence (CDS) of *CiKN6* (Table S5) was cloned into the pBI121 vector. For VIGS assay, tobacco rattle virus (TRV)‐based vectors (pTRV1 and pTRV2) were utilized. To generate the pTRV2‐*CiKN6* construct, a fragment of ~300 bp (from 19 to 319 bp) of the *CiKN6* CDS was cloned into pTRV2. The recombinant vector was then transformed into *Agrobacterium tumefaciens* strain EHA105 using the freeze–thaw method. Primers used are listed in Table S4. The VIGS‐mediated suppression of *CiKN6* expression was performed using a previously reported method (Zeng *et al*., 2021).

Transformation of trifoliate orange (precocious trifoliate orange, *Poncirus trifoliata* L. Raf.) and lemon (*Citrus limon* (L.) Burm.f) was performed using an *Agrobacterium*‐mediated stem segment transformation method, as previously described (Zeng *et al*., 2021). Transgenic plants were confirmed by genomic PCR amplification. The expression levels of *Ci‐miR156c* and *CiKN6* in transgenic trifoliate oranges and lemons were analysed using RT‐qPCR. Total RNA was extracted from leaf samples using the TRIzol Kit (Invitrogen, Carlsbad, CA, USA). First‐strand cDNA was synthesized using the PrimeScript™ II 1st Strand cDNA Synthesis Kit (Vazyme Biotech, Nanjing, China). RT‐qPCR was performed following the manufacturer's instructions for the qPCR SYBR Green Master Mix (Yeasen, Shanghai, China). All primer sequences used for RT‐qPCR analysis are listed in Table S4. Citrus *U6* was used as an internal control to quantify the expression level of *Ci‐miR156c*, whereas citrus *Actin* served as an internal control for the expression levels of other genes. RT‐qPCR analysis was conducted using an Applied Biosystems QuantStudio™ 7 Flex Real‐Time PCR System (ABI, Carlsbad, CA, USA). At least three biological replicates were analysed for each experiment.

### Histological analysis of transgenic plants

The fifth internode (from top to bottom) of the control and transgenic plants was fixed and discoloured using Carnoy's fluid and then treated for transparency using a 3 mol/L NaOH solution. Subsequently, the tissues were washed, dehydrated, waxed, embedded, sectioned, and dewaxed according to a previously reported method (Yao *et al*., 2007). Then, 3–6 μm cross and longitudinal sections from these tissues were dyed with I_2_‐KI to make paraffin sections. Finally, an NIS‐Elements‐B 4.60 microscope (Nikon, Tokyo, Japan) was used for observation. Image J was used to measure the cell diameter and cell number.

### Yeast one‐hybrid assay (Y1H)

For the Y1H assay, the CDSs of nine *CiSPL* genes and *CiKN6* were individually cloned into the pGADT7 vector using the ClonExpress One Step Cloning Kit (Vazyme Biotech). The gene IDs are listed in Table S5. The promoter fragments of *CiFD* (from −166 to −179 bp, three tandem repetitions), *CiKN6* (from −638 to −665 bp), and *CiGA20ox2* (from −1 to −403 bp from −696 to −714 bp) were cloned into the bait vector pAbAi. The primers used are listed in Table S4. The constructed bait vector was transformed into the yeast Y1H‐Gold strain and tested for self‐activation using SD/‐Ura medium containing different concentrations of Aureobasidin A (AbA). Subsequently, nine *CiSPL*‐pGADT7 and *CiKN6*‐pGADT7 vectors were transformed, with the Y1H‐Gold strain containing the bait vector. The interaction screening was performed in SD/‐Leu medium with AbA at 30 °C for 3 days. Competent yeast cells were prepared, and plasmid transformation was performed following the protocol provided in the Matchmaker Gold Yeast One‐Hybrid (Y1H) System Kit (Clontech Laboratories, Inc., Mountain View, CA, USA).

### Dual‐luciferase assay (LUC)

For the LUC assay, promoter fragments of *CiFD*, *CiKN6*, and *CiGA20ox2* were cloned into pGreenII 0800‐LUC to generate a reporter vector. Subsequently, the CDSs of three *CiSPL* genes (*CiSPL3*, *CiSPL6*, *CiSPL7*) and *CiKN6* were inserted into pGreenII 62‐SK under *CaMV35S* promoter control to generate an effector vector. The primers used are shown in Table S4, and the gene IDs are listed in Table S5. The recombinant vectors were then transferred to *Agrobacterium tumefaciens* EHA105. The effector and reporter vectors were transiently co‐expressed in tobacco (*N. benthamiana*) leaves as previously described (Zeng *et al*., 2022). The reporter vector and empty pGreenII 62‐SK vector were co‐transformed into tobacco leaves as a control. A dual‐luciferase reporter assay system (Promega, Madison, Wisconsin, USA) was used to measure the ratio of luminescence of firefly luciferase to renilla luciferase (LUC/REN), according to the manufacturer's instructions.

### Electrophoretic mobility shift assay (EMSA)

For the EMSA assay, ~40 bp promoter fragments (Table S4) from *CiFD*, *CiKN6*, and *CiGA20ox2* containing the SPL binding site were synthesized as 3' biotin‐labelled probes (TSINGKE, Beijing, China). 3' biotin‐labelled mutated oligonucleotide probes and unlabeled probes with the same oligonucleotides were used as mutant and cold competitors. To generate GST‐CiSPL3, GST‐CiSPL6, and GST‐CiSPL7 recombinant vectors, the CDSs of *CiSPL3*, *CiSPL6*, and *CiSPL7* were inserted into the pGEX‐6p‐1 vector. The primers used are shown in Table S4, and the gene IDs are listed in Table S5. The recombinant vectors were transferred into *Escherichia coli* BL21 (DE3) strain. The GST‐CiSPL3, GST‐CiSPL6, and GST‐CiSPL7 proteins were induced by 0.5 mm Isopropyl‐beta‐D‐thiogalactopyranoside (IPTG, BS104 DEXTRA, Guidechem, China). EMSA was performed using an EMSA/Gel‐Shift kit (Beyotime, Shanghai, China) according to the manufacturer's instructions.

### ChIP‐qPCR

*35S*::*CiKN6*‐GFP and *35S*::GFP (control) transgenic citrus plants were subjected to ChIP‐qPCR analysis. The experimental details followed a previously described method (Kaufmann *et al*., 2010). Briefly, ~2 g of leaves was collected from transgenic plants and ground in liquid nitrogen to a fine powder. The samples were then cross‐linked with 1% (v/v) formaldehyde under vacuum for 20 min. The cross‐linking reaction was terminated by adding 1.25 m glycine to the solution, followed by incubation for 5 min. The chromatin was extracted and sonicated using a Bioruptor Plus device. Of the sonicated chromatin, 10% was aliquoted as input controls and stored at −80 °C, while 80% was used for immunoprecipitation (IP) with magnetic beads conjugated to an anti‐GFP Tag mouse monoclonal antibody (ABT2024, Abbkine, Wuhan, China). Both the input and IP DNA were purified using the phenol–chloroform method for subsequent qPCR analysis. The enrichment fold of each fragment was normalized to the internal control, and then by normalizing the value for the IP samples against that for the input. The P1 (−195 to −313 bp), P2 (−531 to −633 bp), P3 (−878 to −990 bp), P4 (−1444 to −1545 bp), and P5 (−1804 to −1893 bp) were located in the promoter of *CiGA20ox2*. All primers used for the ChIP‐qPCR analysis are listed in Table S4.

### Subcellular localization assay

To investigate the subcellular localization of CiSPL3, CiSPL6, and CiSPL7, their CDSs without the stop codon were amplified and fused to the pBI121‐GFP vector, which contained the green fluorescent protein reporter gene under the control of the *CaMV35S* promoter. All primers are listed in Table S4, and the gene IDs are shown in Table S5. The empty vector *35S*::GFP was used as a positive control. Subsequently, the recombinant vectors (*35S*::*CiSPL3*‐GFP, *35S*::*CiSPL6*‐GFP, and *35S*::*CiSPL7*‐GFP) and control vector (*35S*::GFP) were transferred into *Agrobacterium tumefaciens* GV3101 strain. The recombinant or control vectors were transiently expressed in tobacco (*N. benthamiana*) leaves based on a previously described method (Zeng *et al*., 2021). Red fluorescent protein (RFP) was used as a nuclear localization marker and was transiently co‐expressed in tobacco leaves. The infiltrated plants were grown for 2 days in the dark, and fluorescence signals were detected using a laser scanning confocal microscope (TCS‐SP8; Leica, Wetzlar, Germany).

### Transcriptional activity assay

For the transcriptional activity assay, the CDSs of *CiSPL* genes was amplified and inserted into the pGBKT7 vector to fuse with the GAL4‐binding domain (BD). The primers are listed in Table S4, and the gene IDs are shown in Table S5. The recombinant vectors of the pGBKT7‐*CiSPL* and empty vector pGBKT7 were transformed into the yeast AH109 strain. The transformed yeast cells were grown on SD/‐Trp medium for 3 days at 30 °C. Subsequently, the positive clones were cultured in SD/‐Trp, SD/‐Trp/‐His, and SD/‐Trp/‐His medium with the supplement of x‐α‐gal (5‐Bromo‐4‐chloro‐3‐indolyl‐α‐D‐galactoside) and 3 mM 3‐AT (3‐amion‐1,2,4‐trizole) for 3 days at 30 °C. The transcriptional activity of the *CiSPL* genes was identified according to their growth status. For example, the positive yeast cells can grow normally in all the medium and turn blue in SD/‐Trp/‐His medium with the supplement of x‐α‐gal and 3 mm 3‐AT, while the empty vector control (pGBKT7) can only grow in SD/‐Trp. These results suggest that positive yeast cells exhibit transcriptional activate activity.

### RNA‐seq

Total RNA was isolated from the shoot apex of *Ci‐miR156c* transgenic plants and control plants using a TRIzol Kit (Invitrogen) for RNA‐seq, and three biological replicates were performed. mRNA was purified from the total RNA using poly‐T oligo‐attached magnetic beads. Sequencing libraries were generated from purified mRNA using the VAHTS Universal V6 RNA‐seq Library Kit for MGI (Vazyme) following the manufacturer's recommendations with unique index codes. Subsequently, transcriptome sequencing was performed using the MGI‐SEQ 2000 platform to generate 150 bp paired‐end reads by Frasergen Bioinformatics Co., Ltd. (Wuhan, China). To obtain clean reads, raw reads were filtered using the FASTX‐Toolkit (Blankenberg *et al*., 2010) and subsequently mapped to the citrus genome (Chen *et al*., 2014) using the Burrows‐Wheeler Aligner (Li and Durbin, 2009) with default parameters. The NOISeq method was used to screen for DEGs in different samples (Tarazona *et al*., 2015). In this study, the absolute value of log_2_^(fold change)^ ≥ 1.0 and a false discovery rate (FDR) of <0.05 were used as the thresholds. The metabolic pathways of the DEGs were analysed using GO analysis (Mao *et al*., 2005).

### GA content analysis

The shoot apex with some new leaves of *Ci‐miR156c* and *CiKN6* transgenic plants and the controls were collected, and the GA content was determined according to a previously reported method with modifications (Jing *et al*., 2022). Briefly, ~ 200 mg of fresh sample was ground into powder under liquid nitrogen and extracted using 1 mL of an extraction solution containing H_2_O/ACN (acetonitrile) (90:10, v/v). Internal standards were added to the plant samples before extraction. The supernatant was collected by centrifugation at 4 °C for 20 min. The residue was then re‐extracted by repeating the above steps. Then, 10 μL triethylamine and 10 μL 3‐propyl trimethyl ammonium bromide were added to the obtained solution. The reaction solution was vortexed, incubated at 90 °C for 1 h, concentrated to dryness under a stream of nitrogen gas, and redissolved in 100 μL H_2_O/ACN for further liquid chromatograph‐mass spectrometer (LC–MS) analysis. The extracted GA was analysed using an Agilent 1100 HPLC system coupled to an Agilent API3000 mass spectrometer (Agilent Technology, Santa Clara, CA, USA), as described previously (Zeng *et al*., 2021).

### GA_3_ and PAC treatments

For the GA_3_ and PAC treatment assays, GA_3_ and PAC were dissolved in absolute ethanol. Approximately, 3‐month‐old *Ci‐miR156c*‐OE and *Ci‐miR156c*‐STTM transgenic trifoliate oranges, 5‐month‐old *CiKN6*‐OE transgenic lemons, 2‐month‐old *CiKN6*‐VIGS transgenic lemons, and controls were treated. *CiKN6*‐OE and *Ci‐miR156c*‐OE transgenic plants were sprayed with 50 mg/L GA_3_ and 20 mg/L GA_3_, respectively, whereas *CiKN6*‐VIGS and *Ci‐miR156c*‐STTM plants were sprayed with 300 mg/L PAC. The whole plant was sprayed with ~10 mL of GA_3_ or PAC per plant. Under the same growth conditions, all plants were treated once a week for 5 weeks. Compared with the controls (sprayed with the same solution without GA_3_ and PAC), the plant height, internode and leaf number, and average internode length of the treated and untreated plants were measured after 5 weeks.

## Conflicts of interest

The authors declare no conflicts of interest.

## Author contributions

MC, TLZ, WBZ, YZW, ZXM, and ZPX performed the experiments and analysed the data. JZZ and CGH designed the experiments and study. MC wrote the article. JZZ revised the manuscript accordingly. All the authors reviewed and provided comments on the preparation of the manuscript.

## Supporting information


**Figure S1** Bioinformatics analysis of the *Ci‐miR156*.
**Figure S2** Sequence alignment of *miR156* precursors from different plants.
**Figure S3** Phenotypic analysis of *Ci‐miR156a*‐OE transgenic tobacco.
**Figure S4** Functional analysis of *Ci‐miR156b* and *Ci‐miR156c* in tobacco.
**Figure S5** The expression analysis of *Ci‐miR156c* in *Ci‐miR156c* transgenic trifoliate orange.
**Figure S6** Leaf area analysis of *Ci‐miR156c* transgenic trifoliate orange.
**Figure S7** Gene Ontology analysis of differentially expressed genes (DEGs).
**Figure S8** The expression analysis of nine *CiSPL* genes in *Ci‐miR156c* transgenic trifoliate orange.
**Figure S9** The interaction analysis between nine CiSPLs and *CiFT* and *CiFD* through yeast one hybrid assays.
**Figure S10** Subcellular localization and transcriptional activity analysis of CiSPL7 protein.
**Figure S11** The interaction between CiFD and *CiSPL* promoter was analyzed by yeast one‐hybrid assay.
**Figure S12** Expression of *CiKNOX* and analysis of SPL binding sites.
**Figure S13** The interaction between nine CiSPLs and the *CiKN6* promoter was analyzed by yeast one‐hybrid assay.
**Figure S14** Subcellular localization and transcriptional activity analysis of CiSPL3 protein.
**Figure S15** Analysis of *CiKN6* expression in *CiKN6* transgenic lemon.
**Figure S16** The expression of *CiGA20ox2* during plant height development.
**Figure S17** The interaction between nine CiSPLs and *CiGA20ox2* promoter was analyzed by yeast one‐hybrid assay.
**Figure S18** Subcellular localization and transcriptional activity analysis of CiSPL6 protein.
**Figure S19** Transient assays confirmed the *in vivo* interactions between *Ci‐miR156c* and three potential *CiSPL* genes.

**Table S1** Differentially expressed genes from the Ci‐miR156c‐OE vs. the control comparison.
**Table S2** Differentially expressed genes from the Ci‐miR156c‐STTM vs. the control comparison.
**Table S3** Three precursor sequences used in this study.
**Table S4** Primers used in this study.
**Table S5** Gene ID used in this study.

## Data Availability

All data generated or analysed in this study are included in Figures S1–S19 and Tables S1–S5.
