# Supplementary material for: miR156‐SPLs module regulates flowering and controls plant height by modulating gibberellin biosynthesis in citrus
Source: Plant Biotechnol J. 2025 Jun 30;23(10):4271–89. doi: 10.1111/pbi.70238 (PMC12483987; doi:10.1111/pbi.70238)
Supplement: Supplementary file 1 — Figure S1 Bioinformatics analysis of the Ci‐miR156. Figure S2 Sequence alignment of miR156 precursors from different plants. Figure S3 Phenotypic analysis of Ci‐miR156a‐OE transgenic tobacco. Figure S4 Functional analysis of Ci‐miR156b and Ci‐miR156c in tobacco. Figure S5 The expression analysis of Ci‐miR156c in Ci‐miR156c transgenic trifoliate orange. Figure S6 Leaf area analysis of Ci‐miR156c transgenic trifoliate orange. Figure S7 Gene Ontology analysis of differentially expressed genes (DEGs). Figure S8 The expression analysis of nine CiSPL genes in Ci‐miR156c transgenic trifoliate orange. Figure S9 The interaction analysis between nine CiSPLs and CiFT and CiFD through yeast one hybrid assays. Figure S10 Subcellular localization and transcriptional activity analysis of CiSPL7 protein. Figure S11 The interaction between CiFD and CiSPL promoter was analyzed by yeast one‐hybrid assay. Figure S12 Expression of CiKNOX and analysis of SPL binding sites. Figure S13 The interaction between nine CiSPLs and the CiKN6 promoter was analyzed by yeast one‐hybrid assay. Figure S14 Subcellular localization and transcriptional activity analysis of CiSPL3 protein. Figure S15 Analysis of CiKN6 expression in CiKN6 transgenic lemon. Figure S16 The expression of CiGA20ox2 during plant height development. Figure S17 The interaction between nine CiSPLs and CiGA20ox2 promoter was analyzed by yeast one‐hybrid assay. Figure S18 Subcellular localization and transcriptional activity analysis of CiSPL6 protein. Figure S19 Transient assays confirmed the in vivo interactions between Ci‐miR156c and three potential CiSPL genes. [file PBI-23-4271-s002.docx]

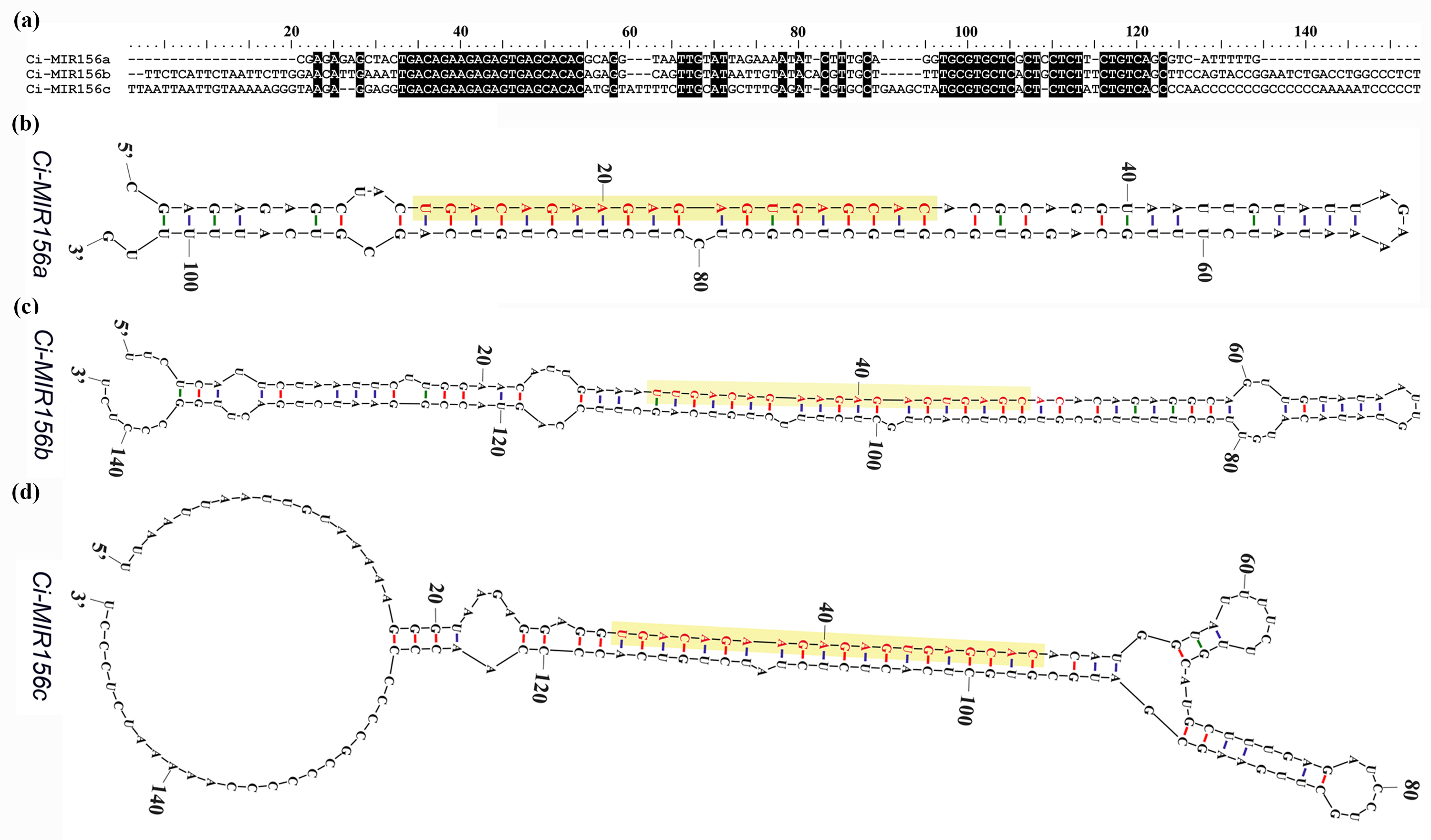


**Figure S1** **Bioinformatics analysis of the *Ci-miR156*.** (a) Sequence alignment of *Ci-MIR156a*, *Ci-MIR156b*, and *Ci-MIR156c* precursors from trifoliate orange. (b-d) The secondary structures of the *Ci-MIR156a* (dG = -49.3 kJ/mol), *Ci-MIR156b* (dG = -57.5 kJ/mol), and *Ci-MIR156c* (dG = -59.5 kJ/mol) precursors were predicted by mfold (http://mfold. rna.albany.edu/?q=mfold). *miR156* mature sequence is highlighted.


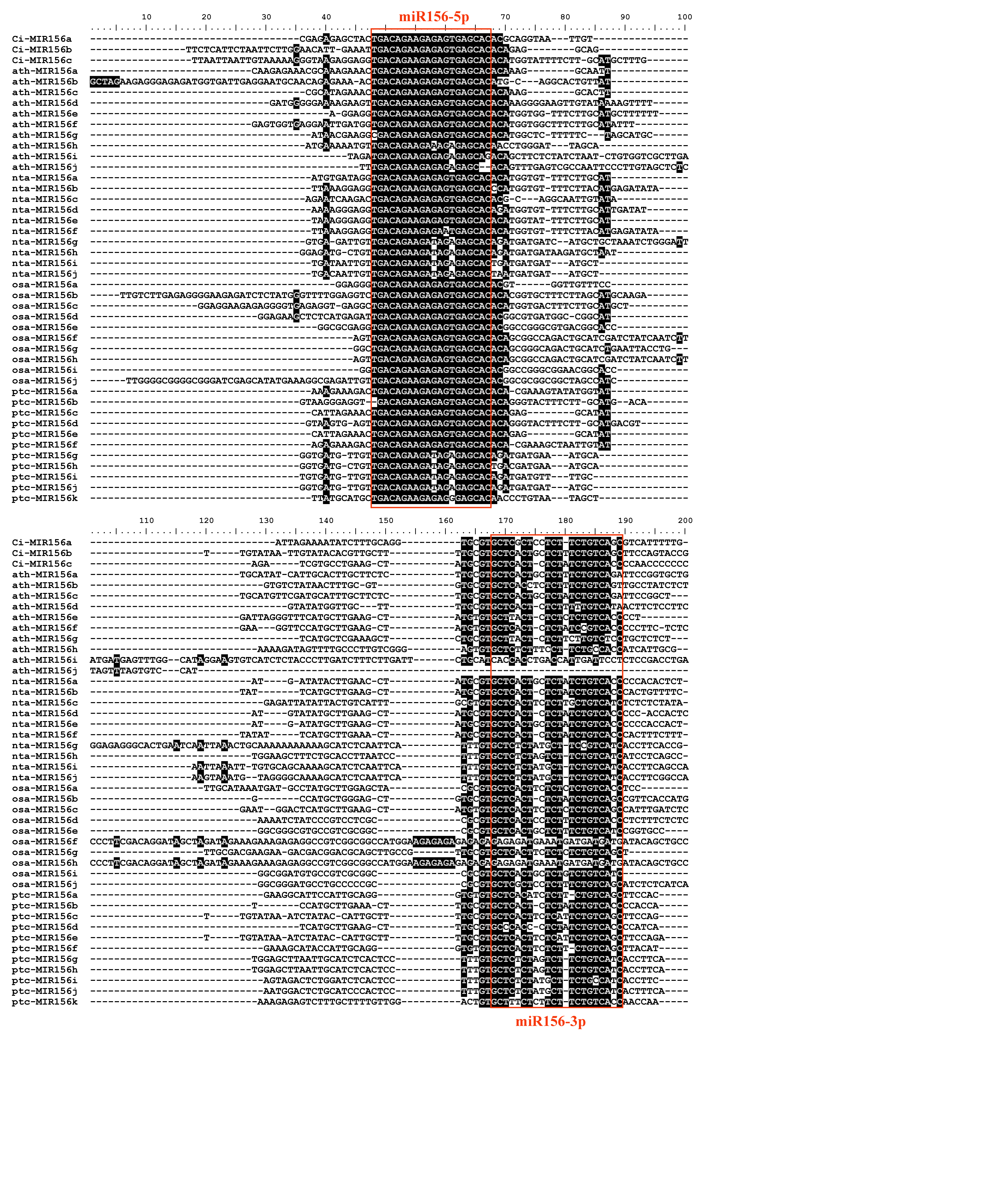


**Figure S2 Sequence alignment of *miR156* precursors from different plants.** *ath-MIR156a*-*ath-MIR156j* from *Arabidopsis* (MI0000178, MI0000179, MI0000180, MI0000181, MI0000182, MI0000183, MI0001082, MI0001083, MI0019232, MI0019234); *nta-MIR156a*-*nta-MIR156j* from *Nicotiana tabacum* (MI0021319, MI0021320, MI0021321, MI0021322, MI0021323, MI0021324, MI0021325, MI0021326, MI0021327, MI0021328); *osa-MIR156a*-*osa-MIR156j* from *Oryza sativa* (MI0000653, MI0000654, MI0000655, MI0000656, MI0000657, MI0000658, MI0000659, MI0000660, MI0000661, MI0000662); *ptc-MIR156a*-*ptc-MIR156k* from *Populus trichocarpa* (MI0002184, MI0002185, MI0002186, MI0002187, MI0002188, MI0002189,) MI0002190, MI0002191, MI0002192, MI0002193, MI0002194). The mature sequences (*miR156*-5p and *miR156*-3p) of *miR156* are highlighted by red boxes.


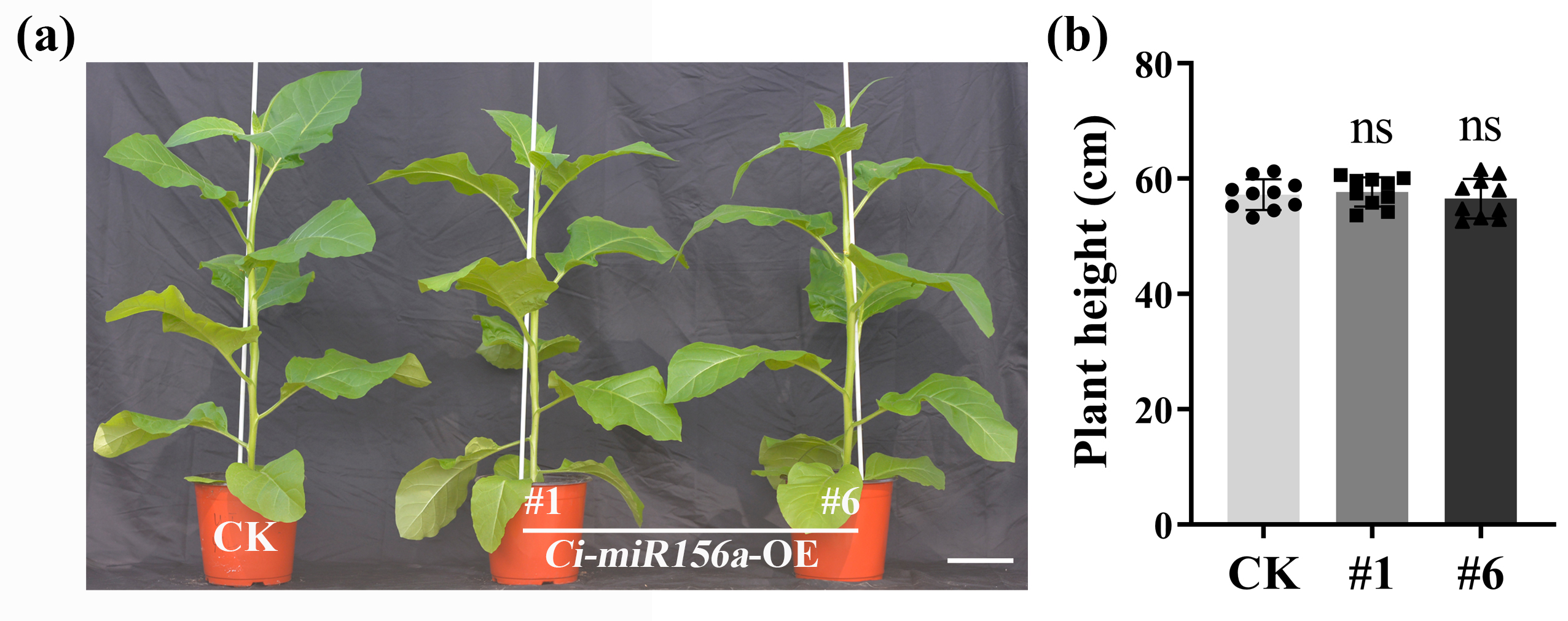


**Figure S3** **Phenotypic analysis of** ***Ci-miR156a*-OE transgenic tobacco****.** (a) Phenotypic analysis of 3-month-old *Ci-miR156a* transgenic tobacco. CK represents the control. #1 and #6 represent two *Ci-miR156a* transgenic lines. Scale bar = 10 cm. (b) Statistical analysis of plant height in *Ci-miR156a* transgenic tobacco and control. Data represent means ± SE (n = 10). Statistically significant differences compared to the control are marked with asterisks (**p* < 0.05, ***p* < 0.01, ns indicates no significant difference, Student’s *t*-test).


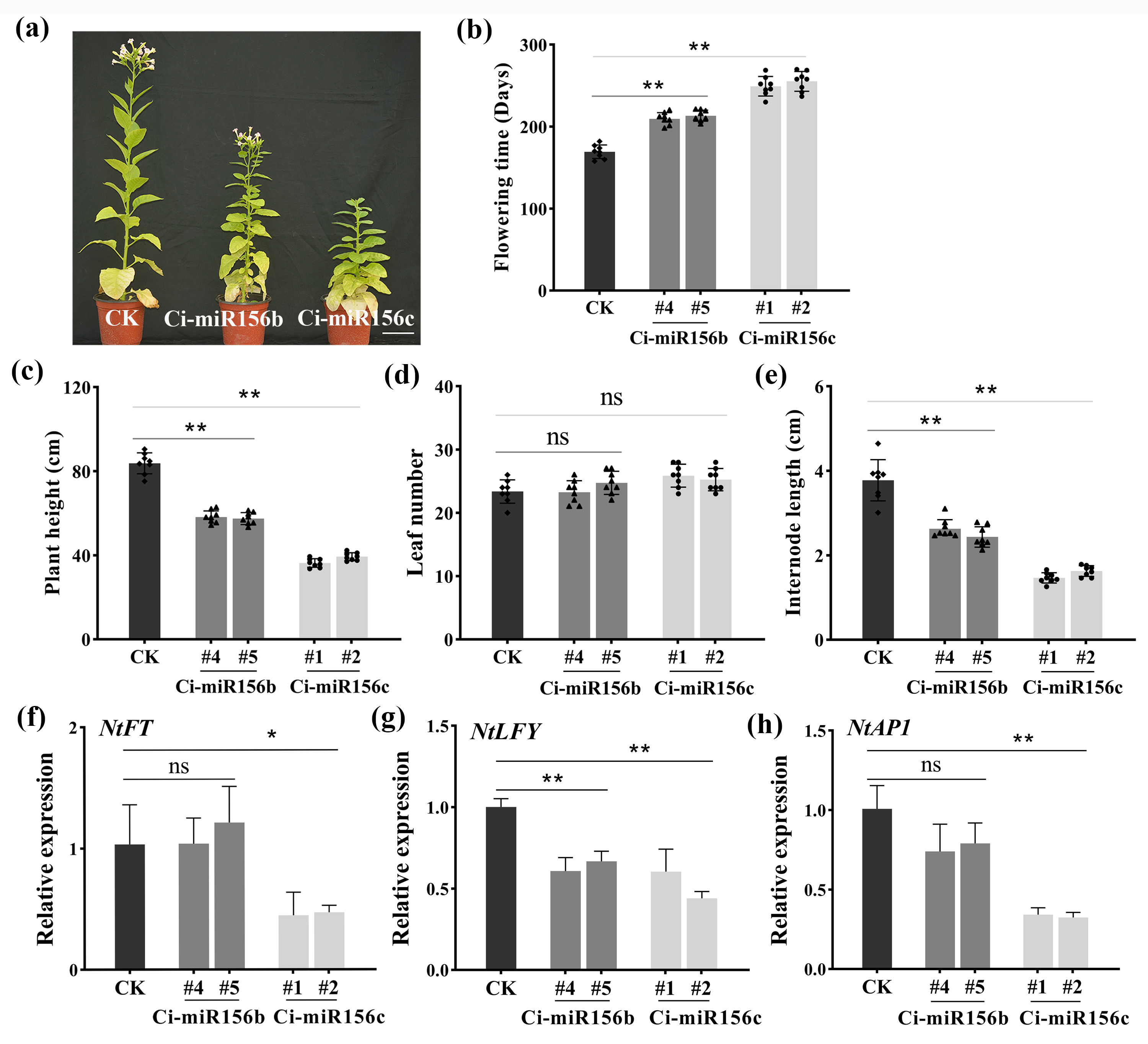


**Figure S4** **Functional analysis of** ***Ci-miR156b* and *Ci-miR156c* in tobacco.** (a) Phenotypic analysis of 7-month-old *Ci-miR156b* and *Ci-miR156c* transgenic tobacco. CK represents the control. Scale bar = 10 cm. (b-e) Statistical analysis of flowering time (b), plant height (c), leaf number (d), and internode length (e) of 8-month-old *Ci-miR156b* and *Ci-miR156c* transgenic tobacco compared with the control. #4 and #5 represent two *Ci-miR156b* transgenic lines. #1 and #2 represent two *Ci-miR156c* transgenic lines. Data represent means ± SE (n = 8). (f-h) The expression analysis of *NtFT* (f), *NtLFY* (g), and *NtAP1* (h) in *Ci-miR156b* and *Ci-miR156c* transgenic tobacco. The shoot apex of 6-month-old tobacco was used for expression analysis. Citrus *Actin* was used as the internal reference gene, and CK was used as the control (with relative expression level set as 1.0). Data represent means ± SE (n = 3). Statistically significant differences compared to the control are marked with asterisks (**p* < 0.05, ***p* < 0.01, ns indicates no significant difference, Student’s *t*-test).


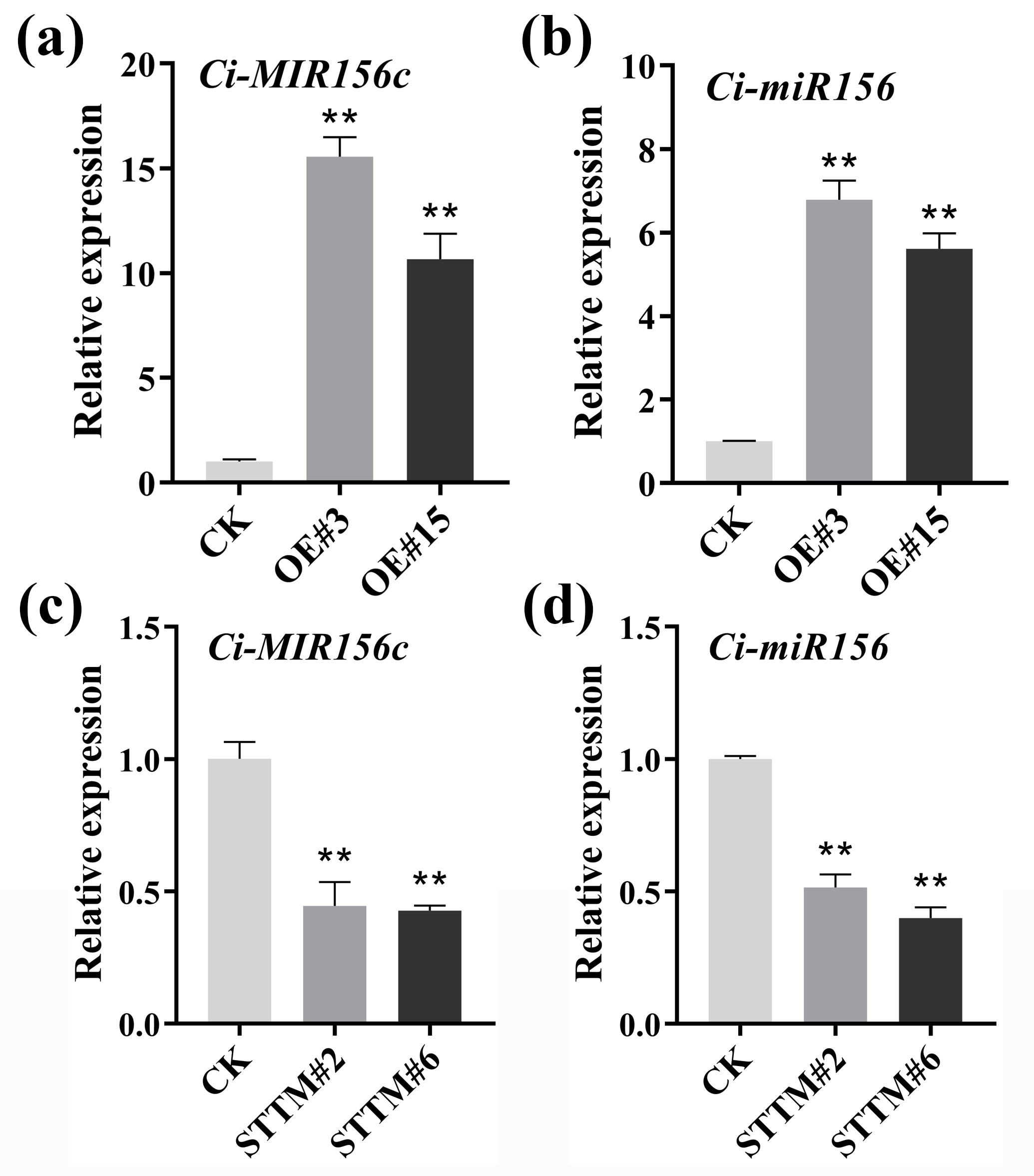


**Figure S5 The expression analysis of *Ci-miR156c* in *Ci-miR156c* transgenic trifoliate orange.** (a-b) The expression analysis of *Ci-MIR156c* (a) and *Ci-miR156* (b) in leaves of 8-month-old *Ci-miR156c*-OE transgenic plants. CK represents the control. OE#3 and OE#15 represent two *Ci-miR156c*-OE transgenic lines. (c-d) The expression analysis of *Ci-MIR156c* (c) and *Ci-miR156* (d) in leaves of 6-month-old *Ci-miR156c*-STTM transgenic plants. CK represents the control. STTM#2 and STTM#6 represent two *Ci-miR156c*-STTM transgenic lines. Citrus *U6* was used as the internal reference gene, and CK was used as the control (with relative expression level set as 1.0). Data represent means ± SE (n = 3). Statistically significant differences compared to the control are marked with asterisks (**p* < 0.05, ***p* < 0.01, ns indicates no significant difference, Student’s *t*-test).


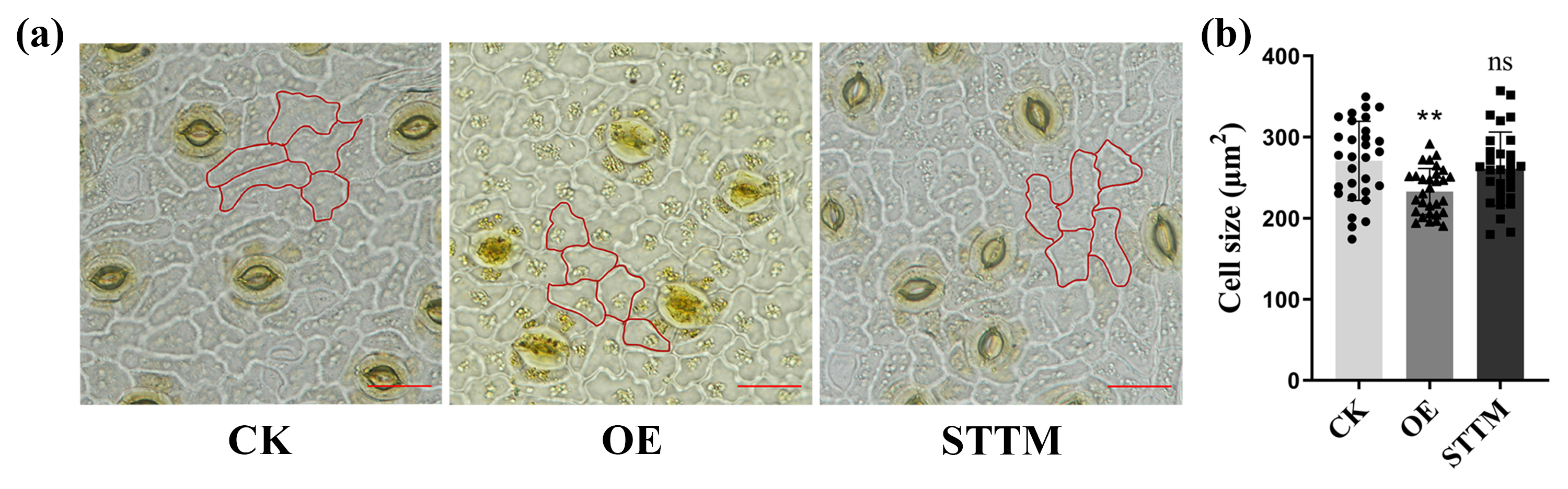


**Figure S6 Leaf area analysis of *Ci-miR156c* transgenic trifoliate orange.** (a) Cytological observation of leaf epidermal cells from the 7th leaf from 6-month-old control plants, *Ci-miR156c*-OE, and *Ci-miR156c*-STTM lines. CK represents the control. OE represents *Ci-miR156c*-OE. STTM represents *Ci-miR156c*-STTM. Five cells are outlined in red to aid visualization. Scale bar = 25 μm. (b) Quantification of the cell size of controls, *Ci-miR156c*-OE, and *Ci-miR156c*-STTM. Data represent means ± SE (n = 30). Statistically significant differences compared to the control are marked with asterisks (**p* < 0.05, ***p* < 0.01, ns indicates no significant difference, Student’s *t*-test).


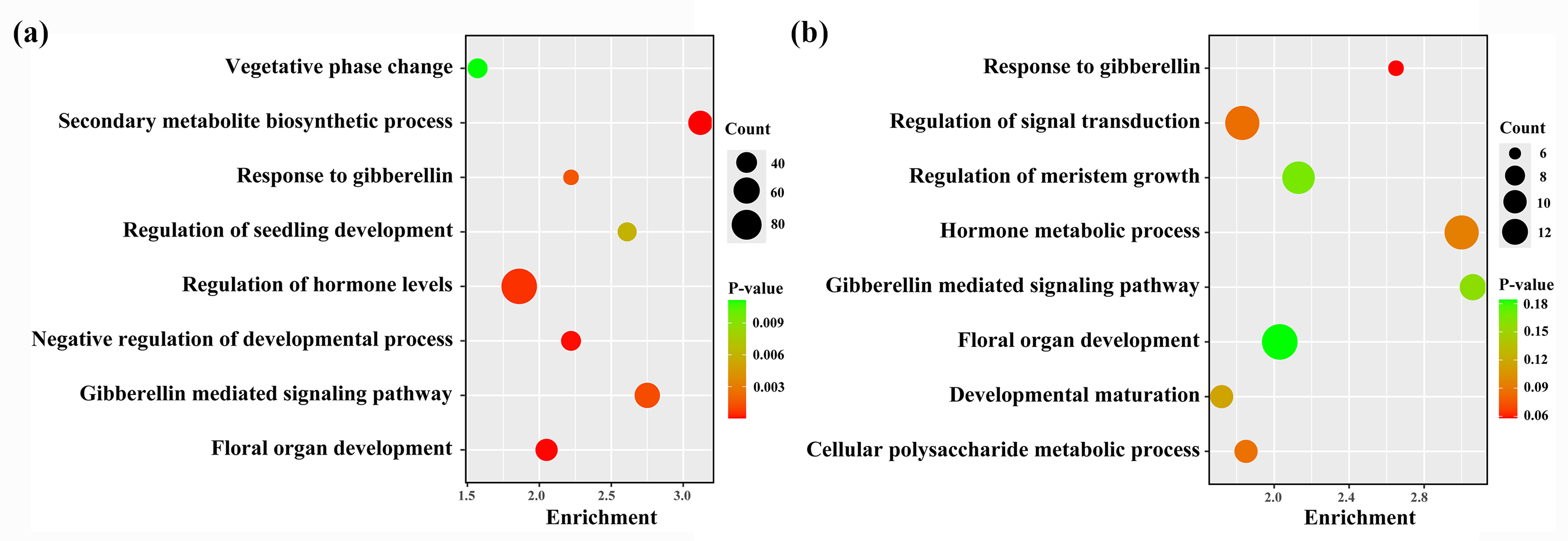


**Figure S7** **Gene Ontology analysis of differentially expressed genes (DEGs).** (a) Biological process analysis of DEGs between *Ci-miR156c*-OE trifoliate orange and the control using Gene Ontology analysis. (b) Biological process analysis of DEGs between *Ci-miR156c*-STTM trifoliate orange and the control using Gene Ontology analysis.

**
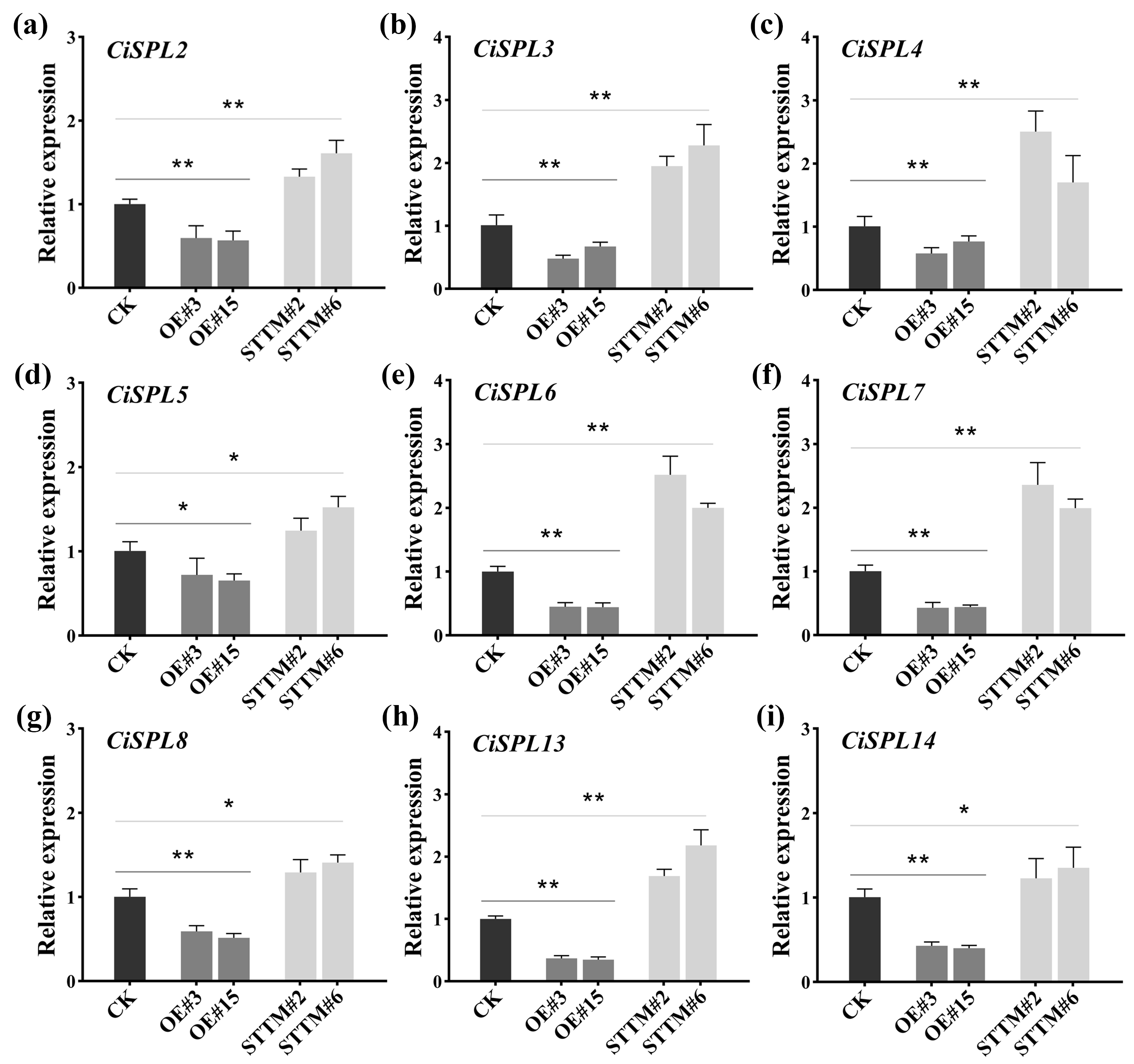
**

**Figure** **S8 The e****xpression analysis of nine *CiSPL* genes in *Ci-miR156c* transgenic** **trifoliate orange.** (a-i) The expression analysis of *CiSPL2* (a), *CiSPL3* (b), *CiSPL4* (c), *CiSPL5* (d), *CiSPL6* (e), *CiSPL7* (f), *CiSPL8* (g), *CiSPL13* (h), and *CiSPL14* (i) in the shoot apex of 6-month-old *Ci-miR156c*-OE and *Ci-miR156c*-STTM transgenic trifoliate orange. CK represents the control. OE#3 and OE#15 represent two *Ci-miR156c-*OE transgenic lines. STTM#2 and STTM#6 represent two *Ci-miR156c*-STTM transgenic lines. Citrus *Actin* was used as the internal reference gene, and CK was used as the control (with relative expression level set as 1.0). Data represent means ± SE (n = 3). Statistically significant differences compared to the control are marked with asterisks (**p* < 0.05, ***p* < 0.01, ns indicates no significant difference, Student’s *t*-test).


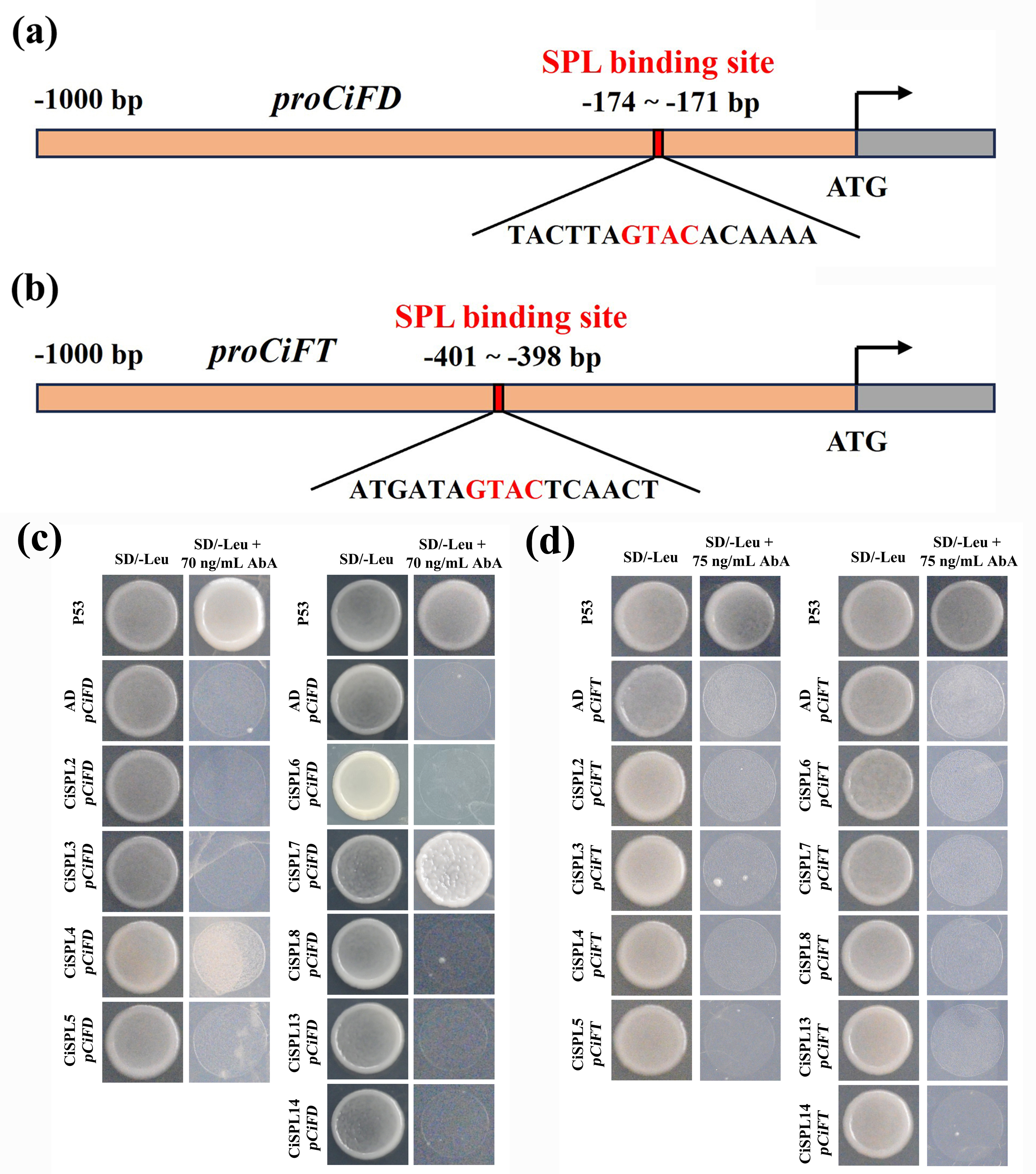


**Figure S9** **The interaction analysis between nine CiSPLs and *CiFT* and *CiFD* through yeast one hybrid assays.** (a) The predicted binding site (GTAC *cis*-element) of CiSPL in the promoter of *CiFD* (from -171 bp to -174 bp) is marked with a red rectangle. (b) The predicted binding site (GTAC *cis*-element) of CiSPL in the promoter of *CiFT* (from -398 bp to -401 bp) is marked with a red rectangle. (c) The interaction analysis between CiSPL and *CiFD* promoter was analyzed by yeast one-hybrid assay. Yeast cells co-transformed with CiSPL7 and the *CiFD* promoter grew well on SD/-Leu plates or SD/-Leu plates supplemented with AbA. P53 was used as the positive control, AD + *pCiFD* was used as the negative control. (d) The interaction analysis between CiSPL and the *CiFT* promoter was analyzed by yeast one-hybrid assay. Yeast cells co-transformed with CiSPL and *CiFT* promoter grew well on SD/-Leu plates but not on SD/-Leu plates supplemented with AbA. P53 was used as the positive control, AD + *pCiFT* was used as the negative control.


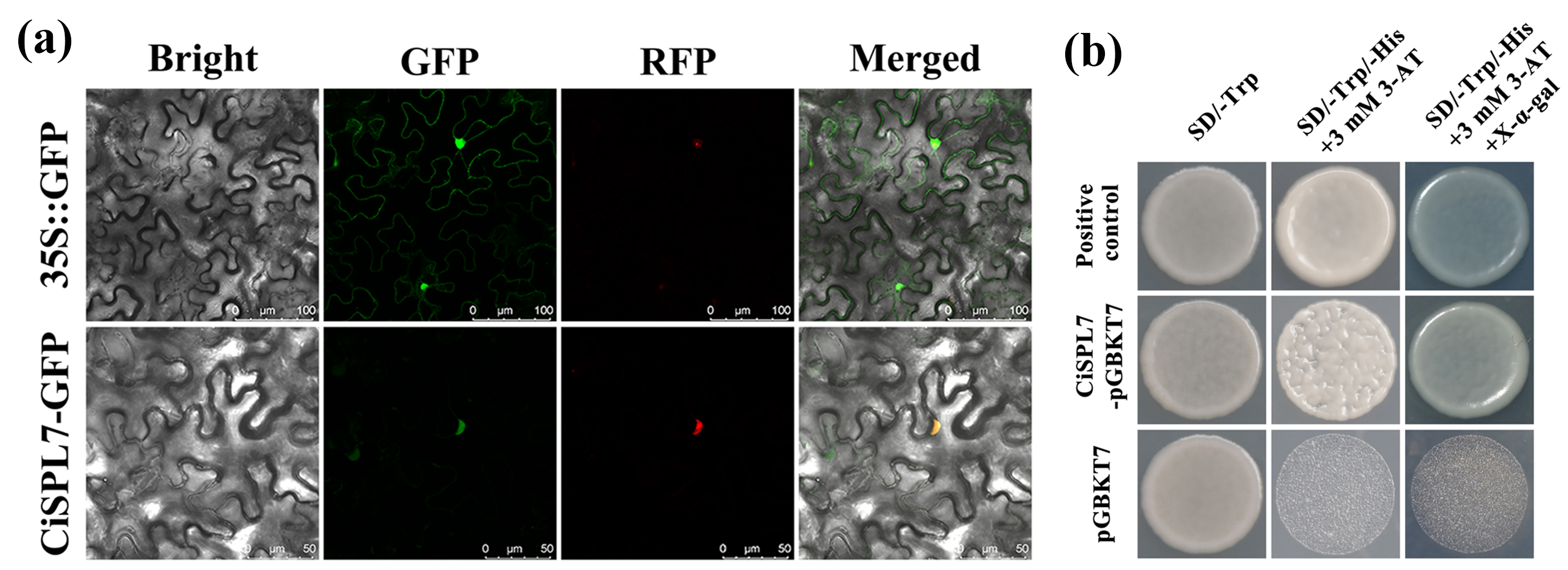


**Figure S10** **Subcellular localization and transcriptional activity analysis of CiSPL7 protein.** (a) Subcellular localization of the CiSPL7 protein in epidermal cells of tobacco leaves transformed using *Agrobacterium* infiltration. The empty vector (*35S*::GFP) was used as a positive control. RFP was used as a nuclear marker. Green color indicates GFP fluorescence. Red color indicates the ﬂuorescence of the nuclear marker (VirD2NLS‐mCherry). GFP, green fluorescent protein. RFP, red fluorescent protein. The scale bar for *35S*::GFP is 100 µm, and the scale bar for CiSPL7-GFP is 50 µm. (b) Transcriptional activity of CiSPL7 in yeast cells. Yeast cells carrying CiSPL7-pGBKT7, the pGBKT7 empty vector (as a negative control) or the positive control were grown on SD/-Trp plates or SD/-Trp/-His plates supplemented with X-α-gal and 3-AT for 3 days at 30 ℃.

**
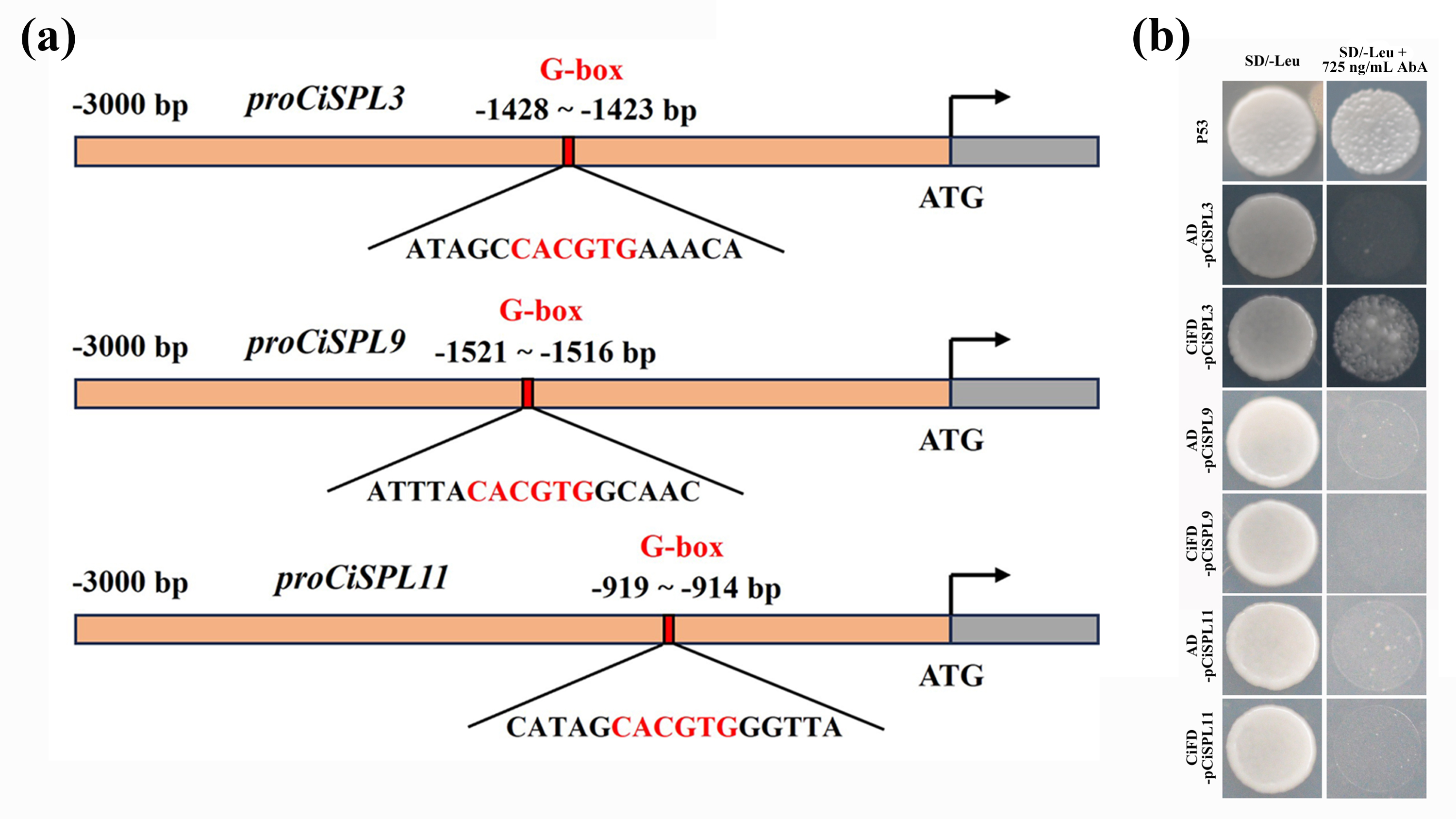
**

**Figure S11 The interaction between CiFD and *CiSPL* promoter was analyzed by yeast one-hybrid assay.** (a) The predicted binding site (G-box element) of CiFD in the promoter of *CiSPL3*, *CiSPL9*, and *CiSPL11* is marked with a red rectangle. (b) Yeast cells co-transformed with CiFD and the *CiSPL3* promoter grew well on SD/-Leu plates or SD/-Leu plates supplemented with AbA. P53 was used as the positive control. AD was used as the negative control.


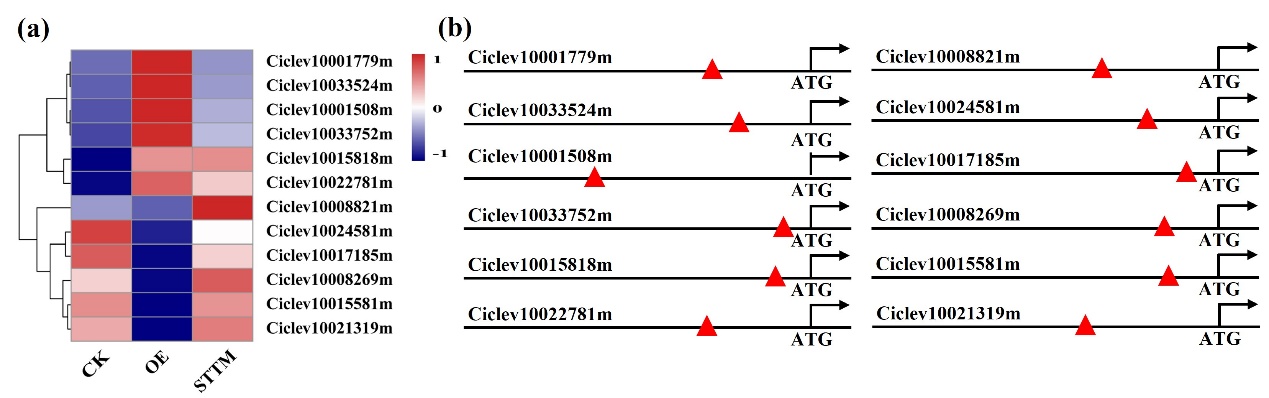


**Figure S12** **Expression of *CiKNOX* and analysis of SPL binding sites** (a) The expression profiles of *CiKNOX* in the control and *Ci-miR156c* transgenic trifoliate orange. CK represents the control. OE represents *Ci-miR156c*-OE transgenic trifoliate orange. STTM represents *Ci-miR156c*-STTM transgenic trifoliate orange. Log_2_ values of FPKM were used to generate the heatmap. Relative expression levels are indicated on a color scale ranging from magenta (high) to blue (low). (b) Analysis of CiSPL binding sites on the *CiKNOX* promoter. The red triangle represents the position of CiSPL binding sites.

**
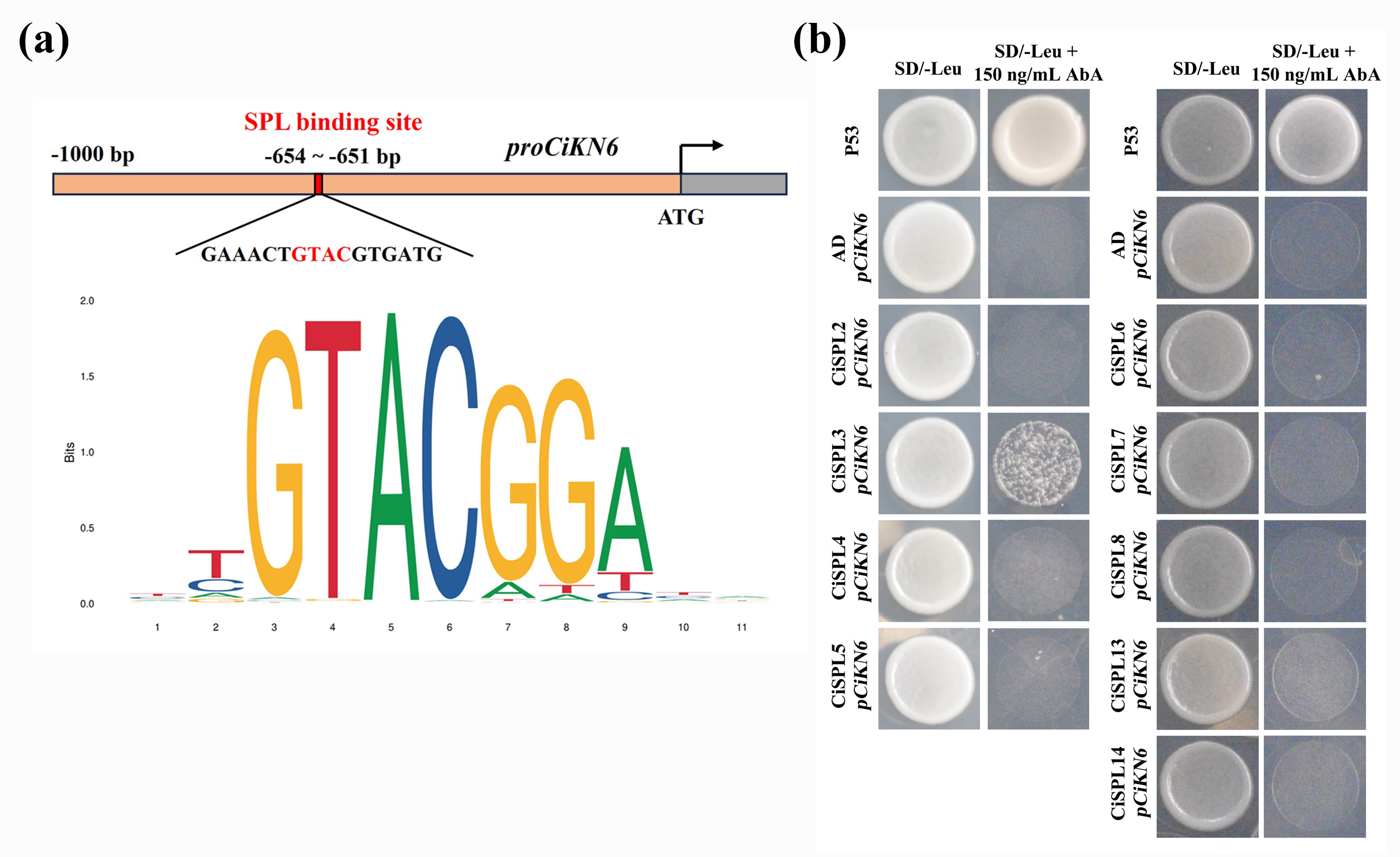
**

**Figure S13** **The interaction between nine CiSPLs and the *CiKN6* promoter was analyzed by yeast one-hybrid assay.** (a) The predicted binding site (GTAC *cis*-element) of CiSPL in the promoter of *CiKN6* (from -651 bp to -654 bp) is marked with a red rectangle. (b) Yeast cells co-transformed with CiSPL3 and the *CiKN6* promoter grew well on SD/-Leu plates or SD/-Leu plates supplemented with AbA. P53 was used as the positive control, AD + *pCiKN6* was used as the negative control.


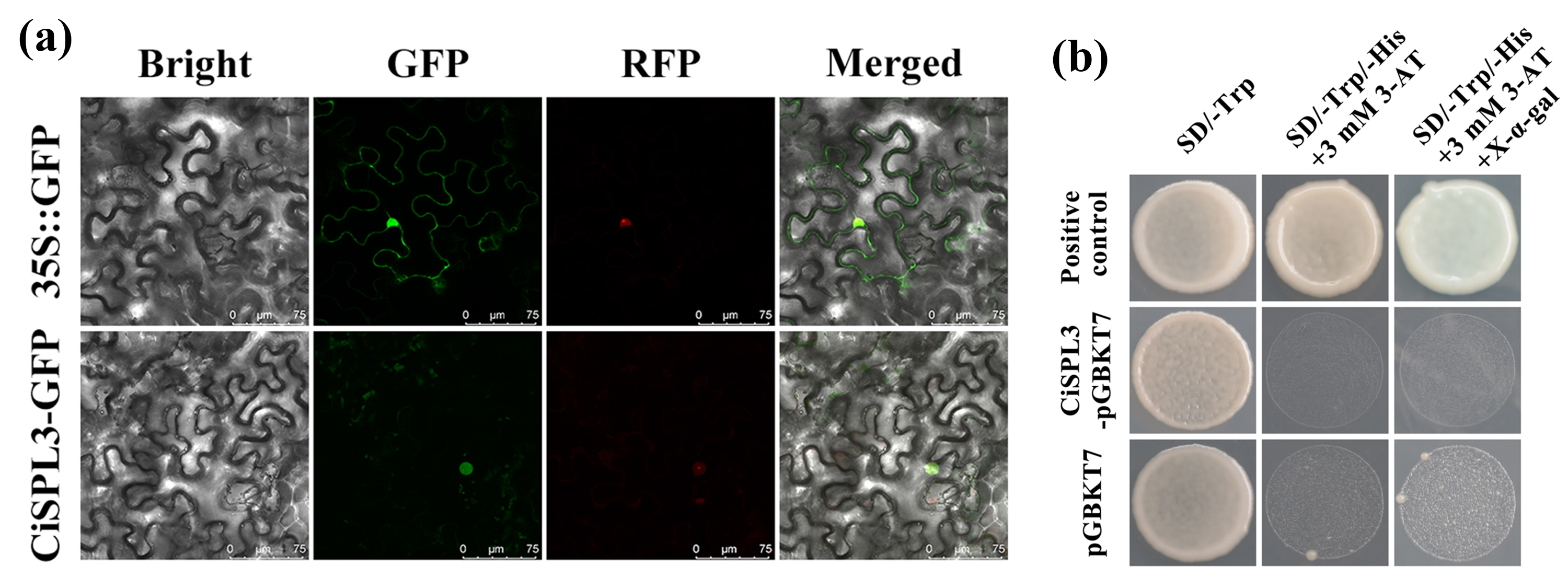


**Figure S14** **Subcellular localization and transcriptional activity analysis of CiSPL3 protein.** (a) Subcellular localization of the CiSPL3 protein in epidermal cells of tobacco leaves transformed using *Agrobacterium* infiltration. The empty vector (*35S*::GFP) was used as a positive control. RFP was used as a nuclear marker. Green color indicates GFP fluorescence. Red color indicates the ﬂuorescence of the nuclear marker (VirD2NLS‐mCherry). GFP, green fluorescent protein. RFP, red fluorescent protein. Scale bar = 75 µm. (b) Transcriptional activity of CiSPL3 in yeast cells. Yeast cells carrying CiSPL3-pGBKT7, the pGBKT7 empty vector (as a negative control) or the positive control were grown on SD/-Trp plates or SD/-Trp/-His plates supplemented with X-α-gal and 3-AT for 3 days at 30 ℃.


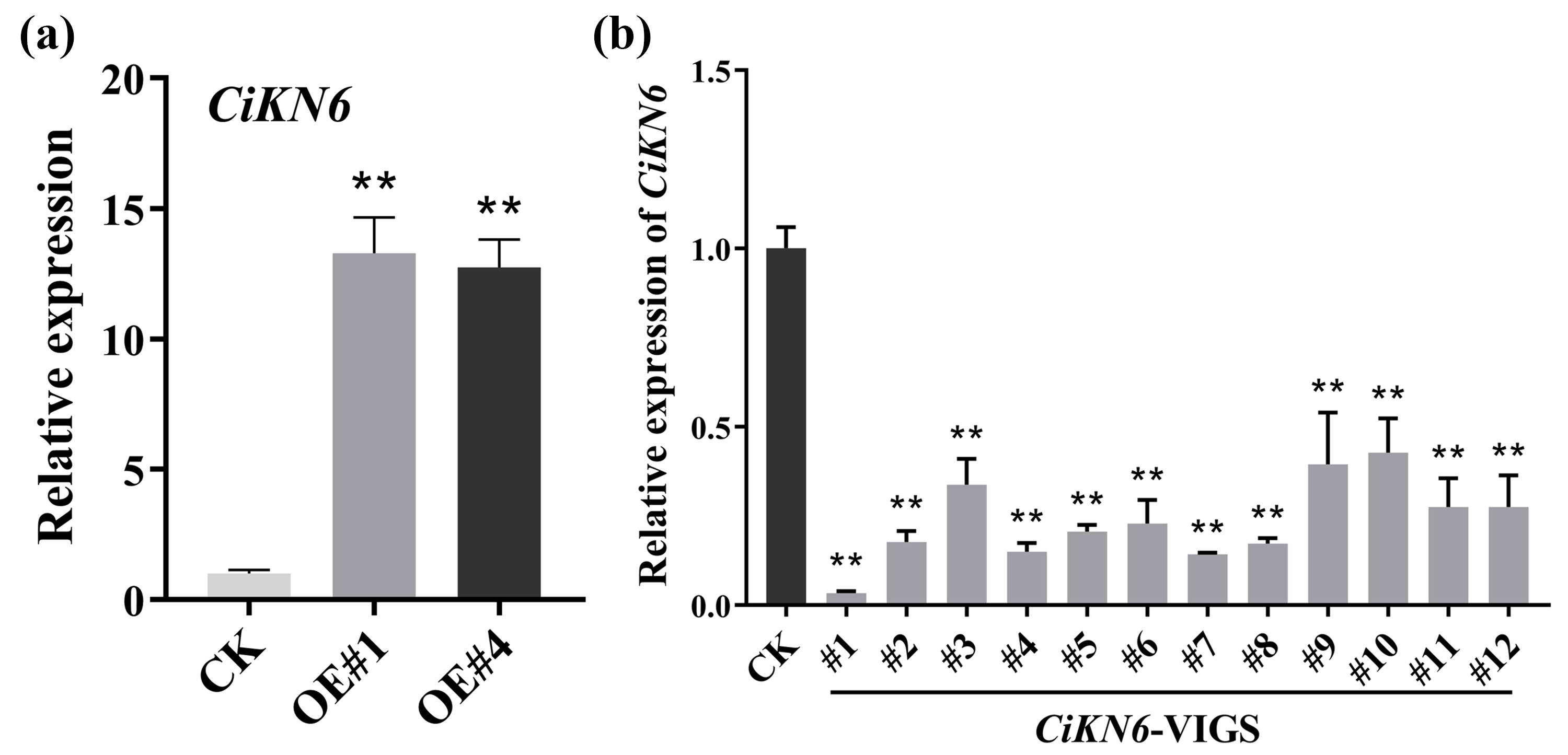


**Figure S15 Analysis of *CiKN6* expression in *CiKN6* transgenic lemon.** (a) The expression analysis of *CiKN6* in leaves of 5-month-old *CiKN6*-OE transgenic lemon. CK represents the control. OE#1 and OE#4 are two selected *CiKN6*-OE transgenic lines. (b) The expression analysis of *CiKN6* in leaves of 3-month-old *CiKN6*-VIGS transgenic lemon. Line #1 to #12 represent twelve *CiKN6*-VIGS lines, respectively. Citrus *Actin* was used as the internal reference gene, and CK was used as the control (with relative expression level set as 1.0). Data represent means ± SE (n = 3). Statistically significant differences compared to the control are marked with asterisks (**p* < 0.05, ***p* < 0.01, ns indicates no significant difference, Student’s *t*-test).


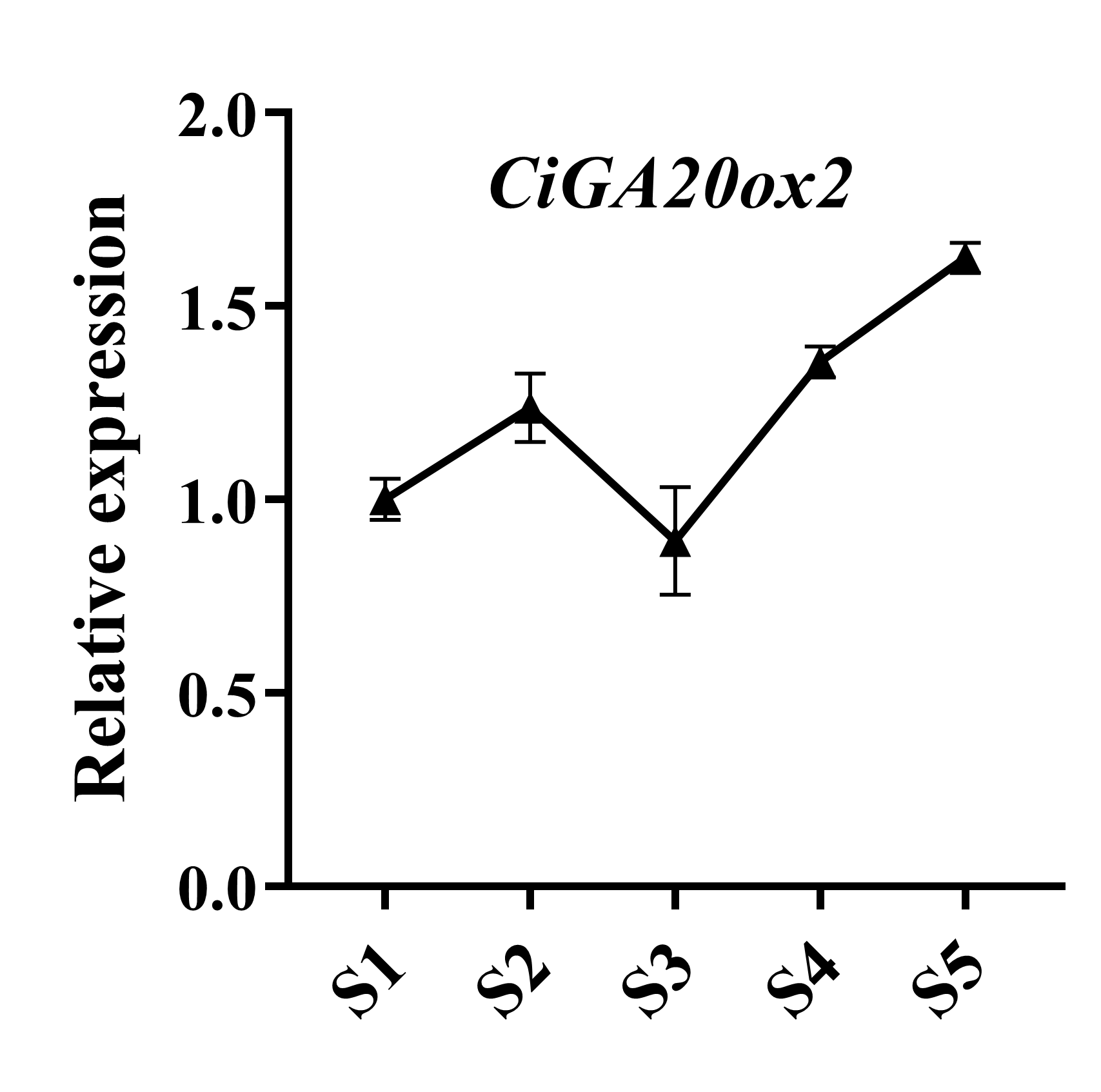


**Figure S16** The expression of *CiGA20ox2* during plant height development. S: stage, S1 to S5 represent different developmental stages with increasing plant height. Citrus *Actin* was used as the internal reference gene, and S1 was used as the control (with relative expression level set as 1.0). Data represent means ± SE (n = 3).

**
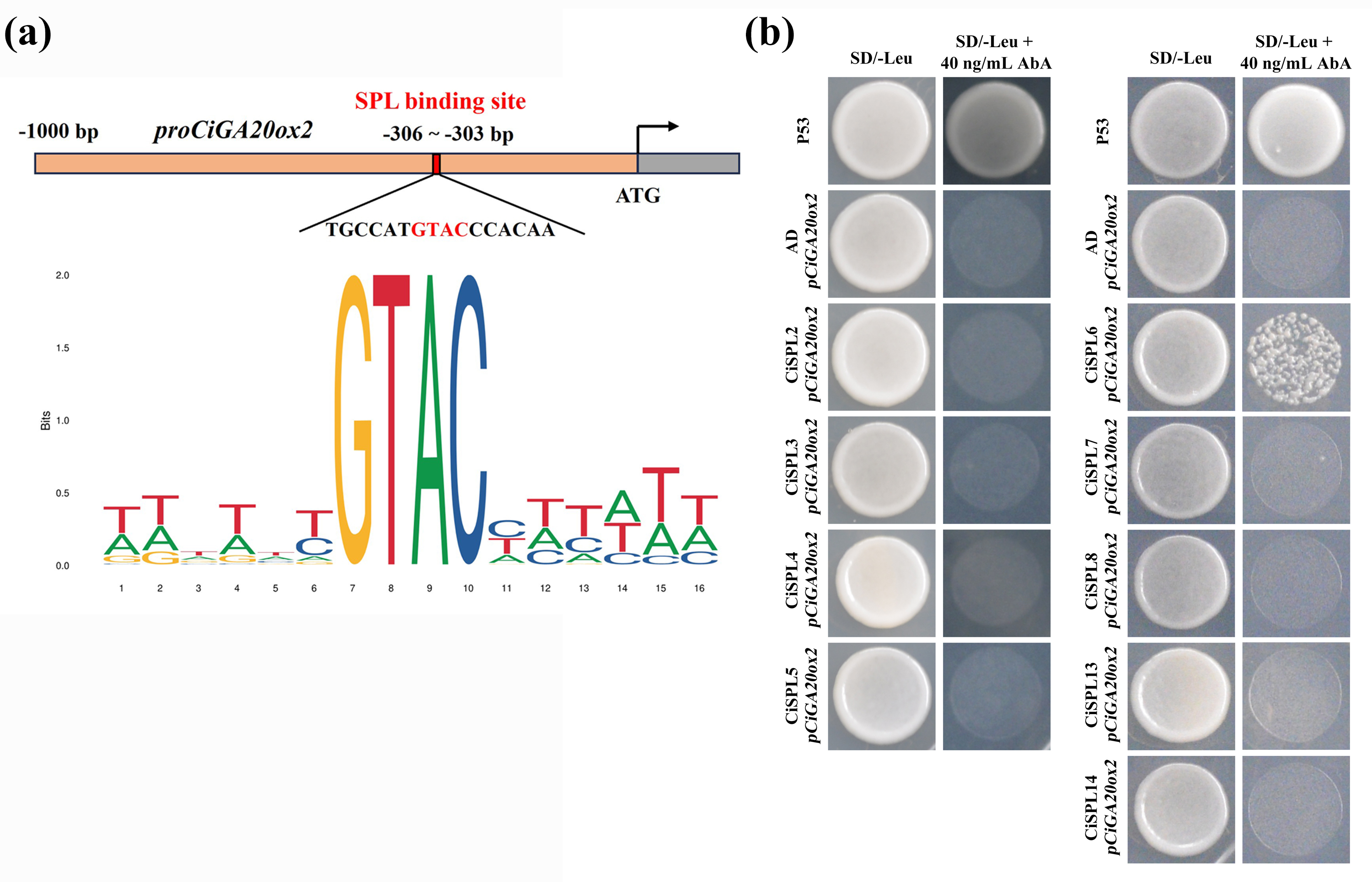
**

**Figure S17** **The interaction between nine CiSPLs and *CiGA20ox2* promoter was analyzed by yeast one-hybrid assay.** (a) The predicted binding site (GTAC *cis*-element) of CiSPL in the *CiGA20ox2* promoter (from -303 bp to -306 bp) is marked with a red rectangle. (b) Yeast cells co-transformed with CiSPL6 and the *CiGA20ox2* promoter grew well on SD/-Leu plates or SD/-Leu plates supplemented with AbA. P53 was used as the positive control, AD + *pCiGA20ox2* was used as the negative control.


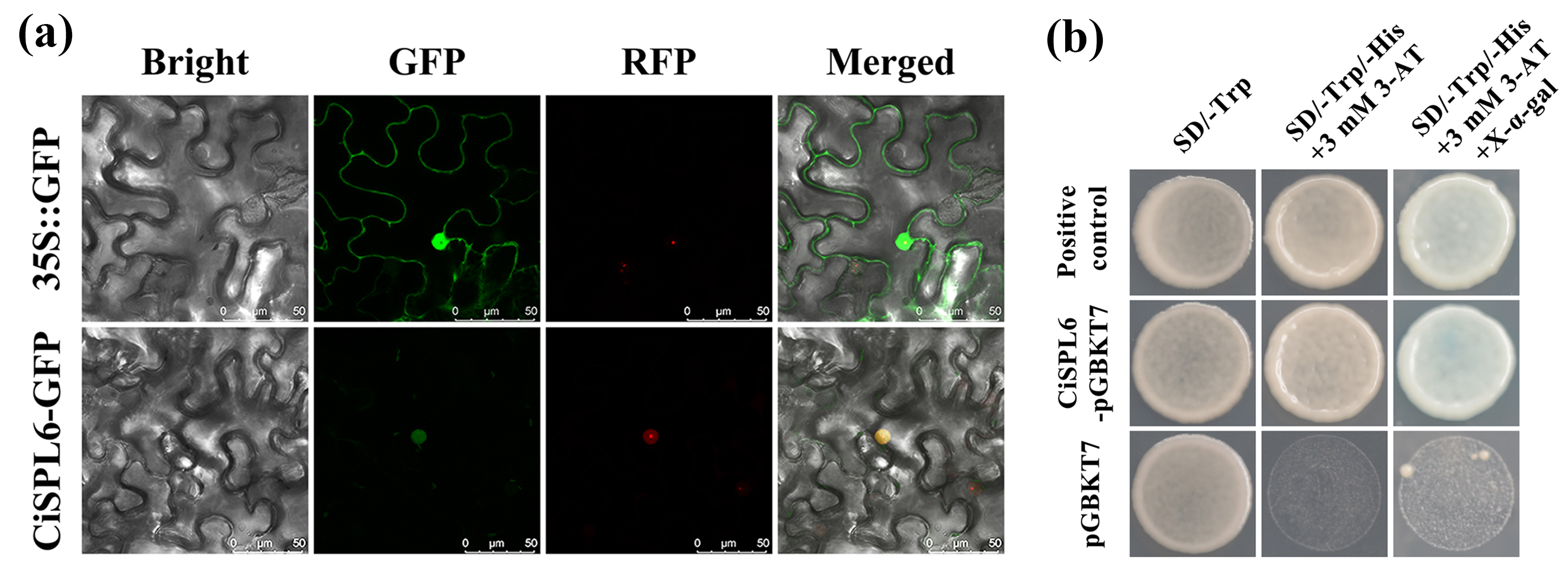


**Figure S18** **Subcellular localization and transcriptional activity analysis of CiSPL6 protein.** (a) Subcellular localization of the CiSPL6 protein in epidermal cells from tobacco leaves transformed using *Agrobacterium* infiltration. The empty vector (*35S*::GFP) was used as a positive control. RFP was used as a nuclear marker. Green color indicates GFP fluorescence. Red color indicates the ﬂuorescence of the nuclear marker (VirD2NLS‐mCherry). GFP, green fluorescent protein. RFP, red fluorescent protein. Scale bar = 50 µm. (b) Transcriptional activity of CiSPL6 in yeast cells. Yeast cells carrying CiSPL6-pGBKT7, the pGBKT7 empty vector (as a negative control) or the positive control were grown on SD/-Trp plates or SD/-Trp/-His plates supplemented with X-α-gal and 3-AT for 3 days at 30 ℃.


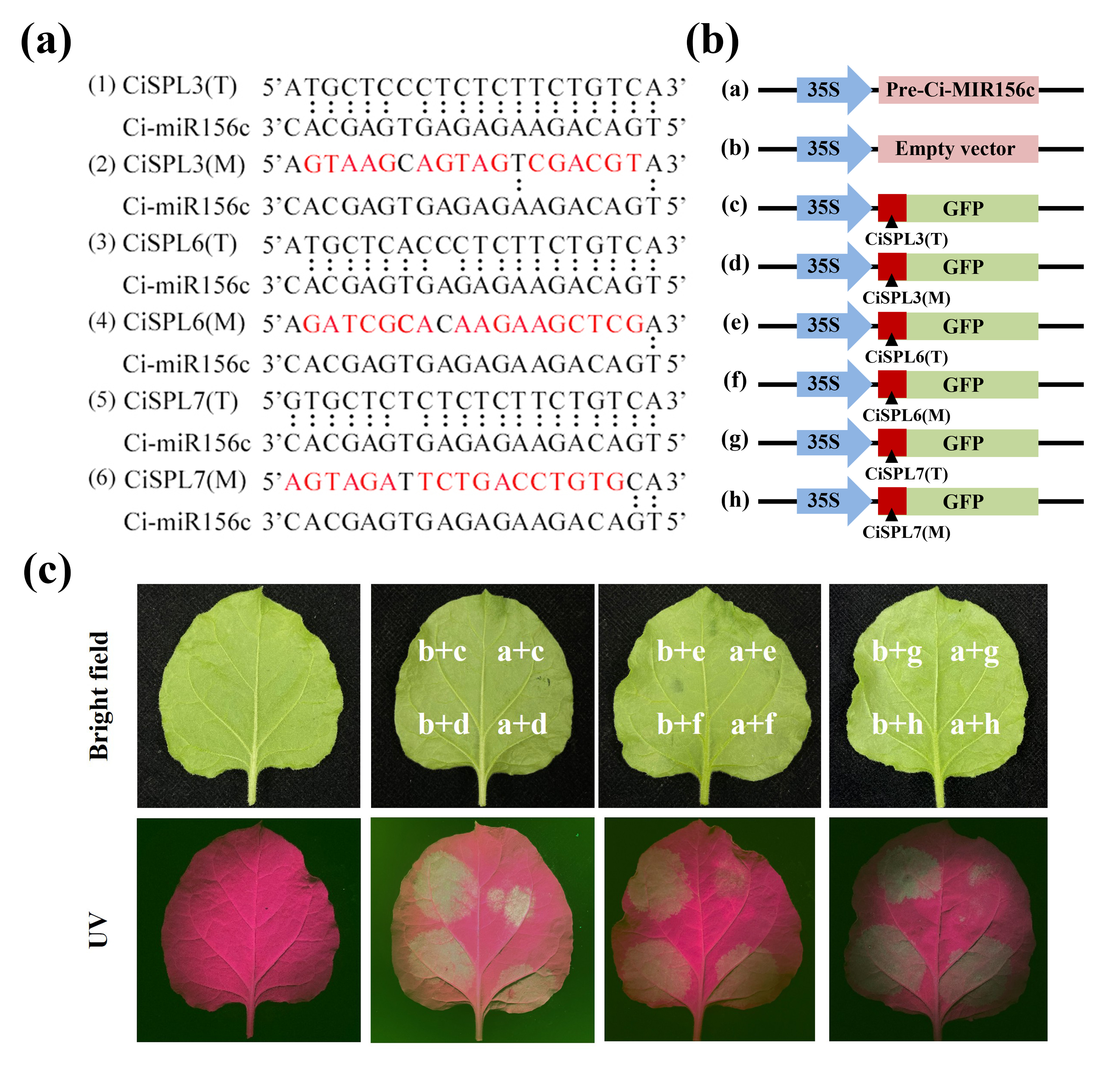


**Figure S19 Transient assays confirmed the *in vivo* interactions between *Ci-miR156c* and three potential *CiSPL* genes.** (a) The sequences of *Ci-miR156c* target sites (T) and mutant sites (M), including of *CiSPL3* (T), *CiSPL3* (M), *CiSPL6* (T), *CiSPL6* (M), *CiSPL7* (T), and *CiSPL7* (M). The double and no point represent perfect and no complementation, respectively. The red nucleotides are modified. (b) Diagrams of the overexpression vectors constructed for transient expression in tobacco leaf. (c) *Agrobacterium tumefaciens* co-injected into tobacco leaf to observe the GFP fluorescence intensity. A reduction of GFP fluorescent signal resulted from co-expression of *Ci-miR156c* with the target site of *CiSPL3* (T), *CiSPL6* (T), and *CiSPL7* (T), compared with the co-expression of empty vector with the target sites. Photos were taken under bright field and UV light (excitation light wavelength 365 nm) 3 d after infection. Wild type leaf without any treatment showed no fluorescence signal under UV.
